# Oncogenic RAS induces a distinctive form of non-canonical autophagy mediated by the P38-ULK1-PI4KB axis

**DOI:** 10.1038/s41422-025-01085-9

**Published:** 2025-03-07

**Authors:** Xiaojuan Wang, Shulin Li, Shiyin Lin, Yaping Han, Tong Zhan, Zhiying Huang, Juanjuan Wang, Ying Li, Haiteng Deng, Min Zhang, Du Feng, Liang Ge

**Affiliations:** 1 State Key Laboratory of Membrane Biology, Beijing, China; 2 Tsinghua-Peking Center for Life Sciences, Beijing, China; 3 School of Life Sciences, Tsinghua University, Beijing, China; 4 Beijing Frontier Research Center for Biological Structure, Beijing, China; 5 School of Pharmaceutical Sciences, Tsinghua University, Beijing, China; 6 State Key Laboratory of Respiratory Disease, Guangzhou Municipal and Guangdong Provincial Key Laboratory of Protein Modification and Degradation, School of Basic Medical Sciences, Guangzhou Medical University, Guangzhou, Guangdong China; 7 MOE Key Laboratory of Bioinformatics, Beijing, China; 8 Department of Anesthesiology, The Second Affiliated Hospital of Guangzhou Medical University, Guangzhou, Guangdong, China; 9 The Affiliated TCM Hospital of Guangzhou Medical University, Guangzhou, Guangdong, China

**Keywords:** Macroautophagy, Tumour biomarkers

## Abstract

Cancer cells with RAS mutations exhibit enhanced autophagy, essential for their proliferation and survival, making it a potential target for therapeutic intervention. However, the regulatory differences between RAS-induced autophagy and physiological autophagy remain poorly understood, complicating the development of cancer-specific anti-autophagy treatments. In this study, we identified a form of non-canonical autophagy induced by oncogenic KRAS expression, termed RAS-induced non-canonical autophagy via ATG8ylation (RINCAA). RINCAA involves distinct autophagic factors compared to those in starvation-induced autophagy and incorporates non-autophagic components, resulting in the formation of non-canonical autophagosomes with multivesicular/multilaminar structures labeled by ATG8 family proteins (e.g., LC3 and GABARAP). We have designated these structures as RAS-induced multivesicular/multilaminar bodies of ATG8ylation (RIMMBA). A notable feature of RINCAA is the substitution of the class III PI3K in canonical autophagy with PI4KB in RINCAA. We identified a regulatory P38-ULK1-PI4KB-WIPI2 signaling cascade governing this process, where ULK1 triggers PI4KB phosphorylation at S256 and T263, initiating PI4P production, ATG8ylation, and non-canonical autophagy. Importantly, elevated PI4KB phosphorylation at S256 and T263 was observed in RAS-mutated cancer cells and colorectal cancer specimens. Inhibition of PI4KB S256 and T263 phosphorylation led to a reduction in RINCAA activity and tumor growth in both xenograft and KPC models of pancreatic cancer, suggesting that targeting ULK1-mediated PI4KB phosphorylation could represent a promising therapeutic strategy for RAS-mutated cancers.

## Introduction

Macroautophagy (henceforth, autophagy) is the massive degradation of intracellular materials and is crucial for maintaining cellular homeostasis and survival under stress conditions.^1,2^ During autophagy, damaged organelles, invasive bacteria, and aggregate-prone proteins are delivered to lysosomes for degradation.^1,3^ Autophagy is relevant in the pathogenesis of diverse diseases, e.g., dysregulated autophagy is associated with cancer.^4–6^ However, the specific molecular events accounting for the dysregulation of autophagy, particularly the differences between cancer-associated autophagy and physiological autophagy, e.g., starvation-induced autophagy, have not been clarified.^7^

The *ras* family of genes, including *hras*, *kras*, and *nras* encode extremely similar 188–189 amino acid proteins and are the most common oncogenes in human cancer.^8^ The *ras* genes encode monomeric GTPases, which function as molecular switches in signal transduction pathways regulating proliferation, differentiation, and survival of mammalian cells.^9^ Mutations that constitutively activate RAS proteins occur in 20%–25% of all human cancers, with a *kras* mutation occurring the most frequently and in multiple cancer types.^10,11^ Activation of a *kras* mutation is a key driver of initiation and progression of many tumors and is essential for tumor growth.^12,13^^,^ However, hyperactive RAS mutants are difficult to target because of the lack of drug-binding pockets on the surface of the RAS proteins.^14^ Until now, the only effective inhibitors for clinical use are AMG510 and MRTX849, which specifically target KRAS(G12C).^15,16^

In addition to directly targeting the RAS proteins, identifying drugs that inhibit downstream RAS effector proteins is a solution for treating cancers with RAS mutations.^17^ It has been shown that activating a RAS mutation associates with the overactivation of autophagy.^17–19^ Studies indicate that RAS-activated autophagy harbors unique features, such as providing nutrients for tumor growth, immune evasion, and remodeling the proteome via selective degradation, e.g., elimination of deleterious inflammatory response pathway components to prevent cytokine-induced paracrine cell death.^20–24^ Accordingly, RAS-mutant tumor cells are very sensitive to autophagy inhibitors, suggesting that autophagy can be exploited as a therapeutic target for RAS-mutant tumors.^25–27^ Because autophagy also maintains cellular homeostasis under physiological conditions, an autophagy inhibitor specifically preventing RAS-induced autophagy without affecting physiological autophagy should be developed. Nonetheless, the regulatory differences between RAS-mutation-induced autophagy and physiological autophagy are unclear, particularly regarding the different involvement of the core autophagy factors, i.e., autophagy-related genes (ATGs) and proteins.

The core steps in autophagy, including biogenesis and maturation of the autophagosome, are regulated by ATGs and other autophagy-related proteins.^28,29^ Under starvation-induced autophagy (a physiological form of autophagy), components of the uncoordinated (UNC)-51-like kinase (ULK) complex, including ULK1/2, ATG13, and RB1 inducible coiled-coil 1 (RB1CC1/FIP200), and ATG101 are activated by inhibiting the mechanistic target of rapamycin complex 1 (mTORC1) and meanwhile activating AMPK.^28,29^ The ULK kinase can phosphorylate multiple autophagy regulators and one major target is the class III phosphoinositide 3 kinase (PI3K) complex comprised of Beclin-1, ATG14, phosphatidylinositol 3 kinase (PIK3) catalytic subunit 3 (PIK3C3/VPS34), PIK3 regulatory subunit 4 (PIK3R4/P150), and activating molecule in Beclin-1-regulated autophagy protein 1 (Ambra1).^28,29^ The PI3K complex phosphorylates phosphatidylinositol (PI) lipids of the phagophore to recruit phosphatidylinositol-3-phosphate (PI3P)-interacting proteins, such as WD repeat domain phosphoinositide-interacting 2 (WIPI2). WIPI2 is an effector of PI3P that activates downstream events by recruiting the lipidation machinery (ATG5–ATG12 conjugate in complex with ATG16L1, ATG3, and ATG7). The lipidation machinery catalyzes the conjugation of microtubule-associated protein 1 light chain 3 (MAP1LC3/LC3/ATG8) to phosphatidylethanolamine (PE), which builds the autophagosome.^28,29^ ATG9A seeds the autophagosome and acts as a scramblase together with ATG2, which transfers lipids to the autophagic membrane.^28,29^ The endosomal sorting complex required for transport (ESCRT) complex seals the phagophore to complete autophagosome biogenesis;^30^ and multiple SNARE complexes together with membrane-tether components facilitate autophagosome-to-lysosome fusion.^29,31,32^ While the cascade and function of ATGs in physiological autophagy are well understood, the regulatory mechanisms governing these autophagic factors under pathological conditions remain less clear, particularly regarding their molecular distinctions as compared with those of physiological autophagy.

Here, we employed multiple oncogenic RAS-induced autophagy models to investigate the underlying molecular mechanisms of autophagy dysregulation in cancer. Our results revealed that KRAS(G12V) induced a non-canonical autophagy featured by ATG8ylation which operates independently of several autophagic factors typically associated with starvation-induced autophagy, such as class III PI3K, ATG9A and ATG2. We term it as RAS-induced non-canonical autophagy via ATG8ylation (RINCAA). Instead of double-membrane autophagosomes, alternative autophagosomes with multivesicular and multilaminar structures positive for ATG8 homologs (e.g., LC3 and GABARAP) were observed. Specifically focusing on the PI3K independence, we discovered that activation of PI4KB by ULK1, induced by the RAS signal, is crucial. Notably, phosphorylation of Peptide 1 on S256 and T263 by RAS-activated ULK1 enhances PI4KB activity, leading to PI4P production. Additionally, similar to starvation-induced autophagy, WIPI2 functions as a downstream effector but acts as a PI4P effector, instead of PI3P, initiating downstream events of autophagosome biogenesis. Inhibition of MEK1/2 and PI4KB phosphorylation on S256 and T263 in combination effectively suppressed autophagy and inhibited the proliferation of RAS-mutant tumor cell lines, and tumor growth in xenograft and LSL-*Kras*^*G12D*^, *p53*^*F/F*^, *Pdx1-Cre* mice (KPC) pancreatic cancer models. These findings elucidate a mechanistic framework for an oncogenic KRAS-induced alternative autophagy and shed lights on a new potent target against cancer with Ras mutations.

## Results

### Establishing a cellular system to analyze KRAS-induced autophagy

**Fig. 1:**
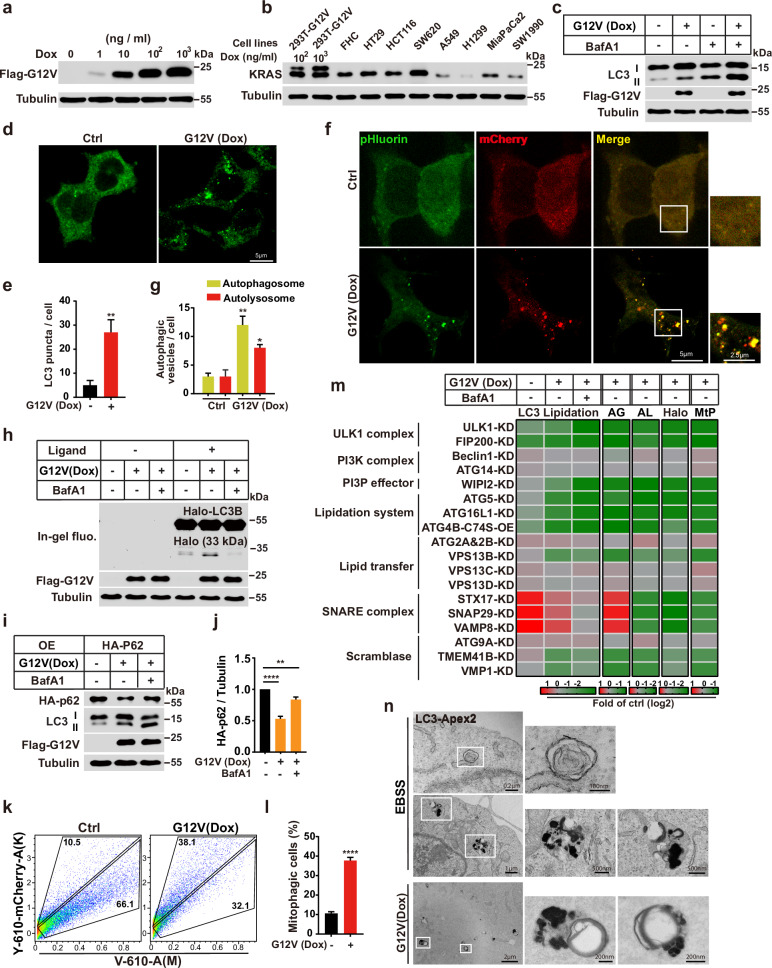
The KRAS(G12V)-induced autophagy system and independency of multiple autophagic factors. **a** Immunoblot analysis of cell lysates derived from KRAS(G12V) HEK293T cells treated with a range of Dox concentrations or left untreated for 36 h. **b** Immunoblot analysis of the expression level of KRAS in different cell lines as indicated. **c** Immunoblot analysis of LC3 lipidation in control and KRAS(G12V) cells with or without 500 nM Baf A1 for 1.5 h. **d** Immunofluorescence and confocal microscopy imaging were performed to visualize the KRAS(G12V)-induced LC3 puncta in HEK293T cells. Representative cell images are shown. Scale bar sizes are indicated in the image. **e** Quantification of the LC3 puncta in control and KRAS(G12V) cells in **d** (mean ± SEM). Three experiments (50 cells for each group/experiment) were performed for the statistics (two-tailed *t*-test). **P < 0.01. **f** Detection of KRAS(G12V)-induced autophagy by the tandem fluorescent LC3 system in control and KRAS(G12V) cells stably expressing mCherry-pHluorin-LC3B. Representative cell images are shown. Scale bar sizes are indicated in the image. **g** Quantification of the yellow (RFP^+^GFP^+^) and Red (RFP^+^GFP^−^) LC3 puncta. Data are presented as mean ± SEM. Three independent experiments (50 cells for each group/experiment) were performed for the statistical analysis (two-tailed *t*-test). * *P* < 0.05; **P < 0.01. **h** Immunoblotting and in-gel fluorescence detection in control and KRAS(G12V) cells stably expressing HaloTag (Halo)-LC3B pulse-labeled for 20 min with 100 nM tetramethyl rhodamine (TMR)-conjugated ligand in nutrient-rich medium with or without 500 nM Baf A1 for 2 h. **i** Immunoblot showing the degradation of exogenous p62 in control and KRAS(G12V) cells with or without 500 nM Baf A1 for 1.5 h. **j** Quantification of the ratio of P62 to tubulin with the control set as 1.00 (control cells without Baf A1) analyzed in **k** (mean ± SEM). Three independent experiments were performed for the statistical analysis (two-tailed *t*-test). **P < 0.01; ****P < 0.0001. **k** Fluorescence-activated cell sorting (FACS) analysis of control and KRAS(G12V) cells co-expressing mt-Keima and Parkin using V610 and Y610-mCherry detectors (Beckman CytoFLEX LX). The FACS results are representative of at least three independent experiments. **l** The percentage of cells with mitophagy based on Y610-mCherry/V610 calculated for **h**. Data are presented as mean ± SEM. Three independent experiments were performed for the statistical analysis (two-tailed *t*-test). ****P < 0.0001. **m** Heatmap showing the changes of LC3 lipidation (the ratio of lipidated LC3 to tubulin with the control set as 1.00, related to Supplementary information, Fig. S1d, e), autophagic flux by the tandem fluorescent LC3 system (autophagosome (AG) and autolysosome (AL), the control set as 1.00, related to Supplementary information, Fig. S1g, h), HaloTag-LC3B processing assay (Halo, Halo-TMR band intensity was normalized by the sum of the band intensities of Halo-TMR-LC3B and Halo-TMR, and the control set (KRAS(G12V) HEK293T cells with ligand without Baf A1) as 1.00, related to Supplementary information, Fig. S1i, g), and mitophagy (MtP, the control set as 1.00, related to Supplementary information, Fig. S1k, l) in control and KRAS(G12V) HEK293T cells with protein knockdown as indicated or ATG4B-C74S overexpression, respectively. Color represents the log_2_ (fold) of counts per samples. **n** Electron microscopy images of APEX2-labeled LC3 and the autophagosomes in Earle’s balanced salt solution (EBSS) or KRAS(G12V) HEK293T cells. Scale bar sizes are indicated in the image.

The expression of a constitutively activated RAS mutant triggers autophagy.^25,33–35^ We were curious to know whether RAS-induced autophagy employs the same cascade of ATGs as starvation-induced autophagy. Therefore, we engineered HEK293T cells to express doxycycline (Dox)-inducible G12V-mutant KRAS (KRAS(G12V)) to activate autophagy. KRAS(G12V) expression was dose dependent and, for subsequent autophagy analysis, we systematically adjusted the concentration and fine-tuned the induction duration to achieve an expression level comparable to that of endogenous RAS in various cancer cell lines (Fig. 1a, b). We examined lipidated LC3 (LC3-II), an autophagic membrane marker. Western blot analysis showed that the Dox treatment increased LC3-II levels both in the presence and absence of the lysosome inhibitor Bafilomycin A1 (Baf A1), which blocks autophagosome maturation, indicating that KRAS(G12V) expression activates autophagy (Fig. 1c). Similar results were obtained in HEK293T cells expressing KRAS(G12D) or KRAS(G12C) alleles (Supplementary information, Fig. S1a). Consistently, LC3 puncta, an indicator of autophagosomes, was increased in Dox-treated KRAS(G12V) cells compared to controls (Fig. 1d, e). In addition, an LC3 paralogue GABARAP colocalized with LC3 in the puncta indicating a general ATG8ylation events on the autophagic membrane (Supplementary information, Fig. S1b).

A double-fluorescence (mCherry-pHluorin) LC3 reporter ^36^was utilized to confirm that KRAS(G12V) enhanced autophagic flux. In this system, autophagosomes (yellow) and autolysosomes (red) were analyzed by fluorescence. Consistent with the effect on autophagosome biogenesis, Dox induction of KRAS(G12V) increased the yellow and red puncta (Fig. 1f, g), indicating that KRAS(G12V) increased both autophagosome formation and autophagic flux. Furthermore, we employed a HaloTag (Halo)-based reporter processing assay to validate the augmented autophagic flux induced by KRAS(G12V) (Fig. 1h).^37^ Subsequently, to corroborate autophagic degradation, we assessed P62 clearance and mitophagy using a mito-keima assay (Fig. 1i–l).^38^ Once more, these findings consistently illustrated an enhanced autophagic turnover following KRAS(G12V) expression. Although the initial protein level of endogenous P62 was increased upon KRAS(G12V) expression, in a cycloheximide chase assay, its lysosome-dependent turnover was increased in the presence of KRAS(G12V) expression further confirming increased autophagic flux (Supplementary information, Fig. S1c, d).

Several inhibitors of KRAS(G12C) have entered clinical trials, including AMG510 (Amgen), which is being evaluated for the treatment of non-small cell lung cancer, colorectal cancer (CRC), and other solid tumors harboring this mutation.^15^ To determine whether autophagy induced by mutant KRAS depends on KRAS activation in our system, we tested the effect of AMG510 on LC3-II. The treatment with AMG510 reduced the levels of LC3-II in cells expressing KRAS(G12C), but not in cells expressing KRAS(G12V) or KRAS(G12D) (Supplementary information, Fig. S1e), suggesting that KRAS activation triggers autophagy upregulation.

Two recent works found that RAS activation may play a negative role on autophagy in multiple KRAS-mutant cancer cells, but MEK inhibition enhanced autophagy in these cells.^39,40^ To clarify the effect of RAS activation on autophagy, we knocked down RAS across six cancer cell lines with RAS mutation and found that it inhibited LC3 lipidation and mitophagy (Supplementary information, Fig. S2a–c), which supports the positive role of RAS activation in autophagy reported by several other studies.^19,20,33–35,41^ Furthermore, trametinib treatment did not notably upregulate autophagy (Supplementary information, Fig. S2a–c).

### Common and distinct utilization of autophagic factors in KRAS(G12V)- and starvation-induced autophagy

To elucidate the role of autophagic factors in oncogenic KRAS-induced autophagy, we utilized gene knockdown to target key autophagic factors involved in various regulatory steps of starvation-induced autophagy in HEK293T cells expressing the Dox-inducible KRAS(G12V) construct. These factors encompassed the ULK kinase complex (ULK1 and FIP200), the PI3K complexes (ATG14, and Beclin-1), the PI3P effector WIPI2, the lipidation machinery (ATG5, ATG16L1, and ATG4B-C74S overexpression), the lipid transfer factors ATG2A and ATG2B, ATG9 vesicles (ATG9A), and the SNARE complexes (STX17, SNAP29, and VAMP8). The knockdown efficiency of each gene was validated by western blot analysis or qPCR (Supplementary information, Fig. S1f, g). Autophagic assays were subsequently employed to comprehensively assess the involvement of these factors in KRAS-induced autophagy.

Consistent with the blockade observed in starvation-induced autophagy, inhibition of the ULK kinase complex, the PI3P effector WIPI2, and the lipidation machinery significantly impaired KRAS(G12V)-induced LC3 lipidation (Fig. 1m; Supplementary information, Fig. S1g, h), and autophagic flux as determined by multiple assays (Fig. 1m; Supplementary information, Fig. S1i–n). Moreover, inhibition of the SNARE complex similarly hindered autophagic flux, aligning with observations in starvation-induced autophagy (Fig. 1m; Supplementary information, Fig. S1g–n). Interestingly, while the PI3K complex, ATG9A, and ATG2s were indispensable for starvation-induced autophagy,^42–51^ their depletion did not impact KRAS-induced autophagy (Fig. 1m; Supplementary information, Fig. S1o–s). Notably, a lipid-transfer protein VPS13B,^52,53^ which functionally transfers lipids akin to ATG2, was essential for KRAS-induced autophagy (Fig. 1m; Supplementary information, Fig. S1g–n). In addition, two scramblases TMEM41B and VMP1 were involved in KRAS-induced autophagy similar to their role in starvation-induced autophagy (Fig. 1m; Supplementary information, Fig. S1g–n).^54–59^ These findings collectively suggest that RAS induces a non-canonical autophagy in which it shares and employs distinct sets of autophagic factors (as well as non-autophagic proteins) compared to starvation-induced autophagy. We term it as RINCAA.

The defined set of autophagic factors orchestrates the generation of double-membrane autophagosomes, a hallmark of starvation-induced autophagy. We investigated whether similar double-membrane autophagosomes could be generated with altered autophagic factors. An APEX2 labeling assay, combined with electron microscopy (EM), was performed to determine the structure of APEX2-LC3-labeled membranes. Interestingly, instead of double-membrane autophagosomes generated in starvation-induced autophagy (Fig. 1n), multivesicular and multilaminar structures of LC3 were identified in KRAS(G12V)-expressing cells and multiple cancer cell lines with RAS mutations (Fig. 1n; Supplementary information, Fig. S1t). Notably, irregular multivesicular and multilaminar (occasionally, data not shown) structures of LC3 were also observed in starvation conditions. We consider them as structures in late stages such as amphisomes or autolysosomes (Fig. 1n). On the contrary, RAS-induced multivesicular and multilaminar structures are likely early stage structures equal to the double-membrane autophagosomes (Fig. 3e; Supplementary information, Video S1). Thus, activated KRAS mutations induce the generation of alternative autophagosomes using a different set of autophagic factors. We term these alternative autophagosomes RAS-induced multivesicular and multilaminar bodies positive of ATG8ylation (RIMMBA).

### PI4KB regulates RINCAA

The intriguing observation of PI3K independence in RINCAA piqued our interest, as the PI3K complex is traditionally considered pivotal in linking protein signaling to membrane remodeling processes in starvation-induced autophagy. Despite the dispensability of PI3K, we found that the PI3P effector WIPI2 remains indispensable in this context (Fig. 1m; Supplementary information, Fig. S1g–n). Given WIPI2’s potential ability to bind various types of PI-phosphates, we hypothesized that another PI-kinase might play the role of PI3K in KRAS-induced atg8ylation of RINCAA. To identify the specific PI-kinase(s) and PI-phosphates involved, we assessed the impact of different phosphokinase inhibitors on LC3-II levels in KRAS(G12V)-induced HEK293T cells.

**Fig. 2:**
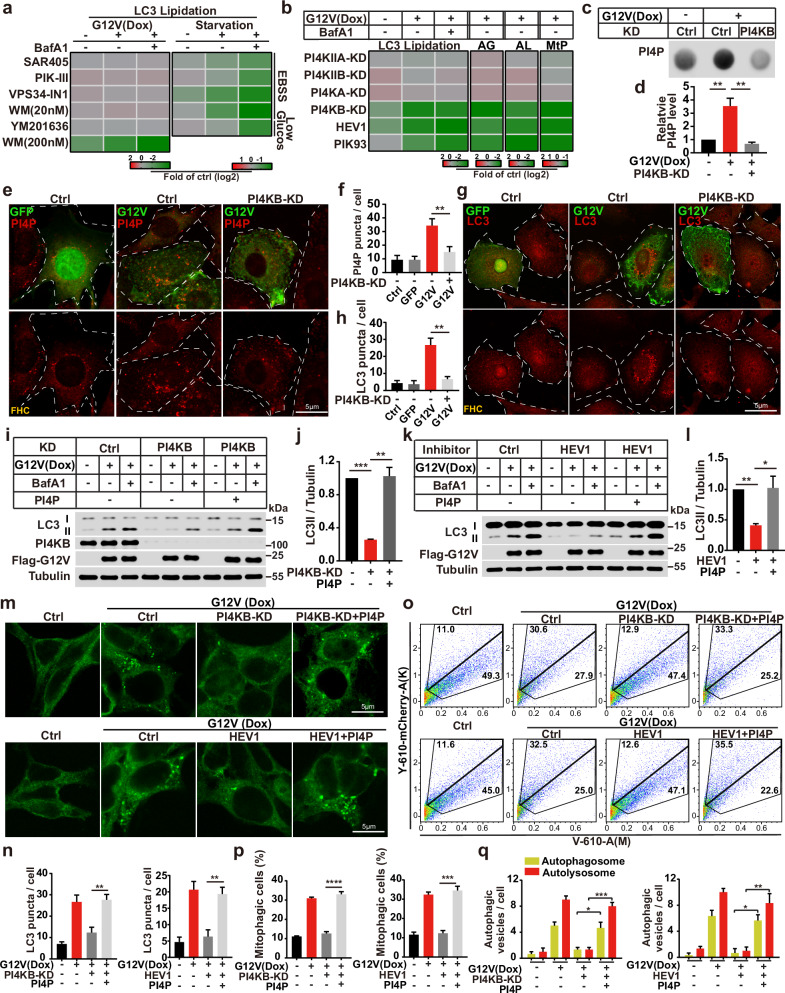
PI4KB regulates RINCAA. **a** Heatmap showing the changes of LC3 lipidation (the ratio of lipidated LC3 to tubulin with the control set as 1.00) in KRAS(G12V) and starvation groups with different inhibitors (related to Supplementary information, Fig. S3a−e). Color represents the log_2_ (fold) of counts per samples. **b** Heatmap showing the changes of LC3 lipidation (the ratio of lipidated LC3 to tubulin with the control set as 1.00, related to Supplementary information, Fig. S3h, i), autophagic flux (AG and AL, related to Supplementary information, Fig. S3g, k) and mitophagy (MtP, related to Supplementary information, Fig. S3l, m) in KRAS(G12V) HEK293T cells with knockdown of PI4Ks or treatment of PI4KB inhibitors. Color represents the log_2_ (fold) of counts per samples. **c** Dot blot analysis of the KRAS(G12V)-induced PI4P generation by knocking down PI4KB in control and KRAS(G12V) HEK293T cells. **d** Quantification of results shown in **c** (mean ± SEM), and the control set as 1.00. Three independent experiments were performed for the statistical analysis (two-tailed *t*-test). **P < 0.01. **e** Immunofluorescence analysis of the PI4P puncta in KRAS(G12V) FHC cells with or without PI4KB knockdown. Representative cell images are shown. Scale bar sizes are indicated in the image. **f** Quantification of the results shown in **e** (mean ± SEM). Three independent experiments (50 cells for each group/experiment) were performed for the statistical analysis (two-tailed *t*-test). **P < 0.01. **g** Immunofluorescence and confocal microscopy imaging were performed to visualize the KRAS(G12V)-induced LC3 puncta in FHC cells with or without PI4KB knockdown. Representative cell images are shown. Scale bar sizes are indicated in the image. **h** Quantification of the LC3 puncta in control and KRAS(G12V) FHC cells in **g**. Data are represented as mean ± SEM. Three independent experiments (50 cells for each group/experiment) were performed for the statistical analysis (two-tailed *t*-test). **P < 0.01. **i**−**l** Immunoblot analysis of the LC3 lipidation rescued by PI4P in KRAS(G12V) HEK293T cells with suppressed PI4KB by knockdown (**i**, **j**) or treatment of the inhibitor (**k**, **l**). Quantification of the results in **i**, **k** are shown in **j**, **l** (mean ± SEM), respectively. Three independent experiments were performed for the statistical analysis (two-tailed *t*-test). *P < 0.05; **P < 0.01; ***P < 0.001. **m** Immunofluorescence analysis of the LC3 puncta rescued by PI4P in KRAS(G12V) HEK293T cells with suppressed PI4KB by knockdown or treatment of the inhibitor. Representative cell images are shown. Scale bar sizes are indicated in the image. **n** Quantification of the results shown in **m** (mean ± SEM). Three independent experiments (50 cells for each group/experiment) were performed for the statistical analysis (two-tailed *t*-test). **P < 0.01. **o** FACS analysis of mitophagy rescued by PI4P in KRAS(G12V) HEK293T cells with suppressed PI4KB by knockdown or treatment of the inhibitor. The FACS results are representative of at least three independent experiments. **p** The percentage of cells with mitophagy based on Y610-mCherry/V610 calculated for **o**. Error bars represent standard deviations of three experiments (mean ± SEM). Three experiments were performed for the statistical analysis (two-tailed *t*-test). ***P < 0.001; ****P < 0.0001. **q** Quantification of the yellow (RFP^+^GFP^+^) and Red (RFP^+^GFP^−^) LC3 puncta (related to Supplementary information, Fig. S3m). KRAS(G12V) HEK293T cells were rescued by PI4P with suppressed PI4KB by knockdown or treatment of the inhibitor. Data are presented as mean ± SEM. Three independent experiments (50 cells for each group/experiment) were performed for the statistical analysis (two-tailed *t*-test). *P < 0.05; **P < 0.01; ***P < 0.001.

Remarkably, treatment with PI3K inhibitors SAR405,^60^ PIK-III,^61^ VPS34-IN1,^62,63^ and wortmannin^64^ at 20 nM did not reduce LC3-II levels in Dox-induced cells compared to the control, nor did the PI5K inhibitor YM201636^65^ (Fig. 2a; Supplementary information, Fig. S3a, b). Conversely, these inhibitors effectively attenuated starvation-induced and glucose starvation-induced autophagy (Fig. 2a; Supplementary information, Fig. S3c–e). Intriguingly, treatment with 200 nM wortmannin, which inhibits type III PI4Ks,^66^ led to decreased LC3-II levels during Dox treatment (Fig. 2a; Supplementary information, Fig. S3b–f), suggesting potential involvement of PI4K and PI4P in RINCAA.

We evaluated the LC3-II levels and performed autophagic flux assays in our system with shRNA-mediated knockdown of PI4K isoforms to identify which PI4K is the main regulator of RINCAA. The knockdown efficiency of all isoforms was > 80% (Supplementary information, Fig. S3g). Knockdown of PI4KIIA, PI4KIIB, and PI4KA did not affect KRAS(G12V)-induced LC3-II accumulation, whereas PI4KB knockdown resulted in marked depletion of LC3-II and autophagic flux (Fig. 2b; Supplementary information, Fig. S3h–m). We examined the effect of the PI4KB inhibitors on RINCAA to confirm the effects of PI4KB. As a result, T-00127-HEV1^67^ and PIK93^68,69^ robustly inhibited KRAS(G12V)-induced LC3 lipidation and autophagic flux (Fig. 2b; Supplementary information, Fig. S3h–m). The dependence on PI4KB in autophagic flux was also confirmed by the P62 turnover assay as described above (Supplementary information, Fig. S3n, o).

Furthermore, we assessed the PI4P levels using dot blot and PI4P staining assays and found that KRAS(G12V) expression increased PI4P levels which was abrogated by PI4KB knockdown (Fig. 2c–f). Similarly, PI4KB silencing or T-00127-HEV1 treatment markedly reduced KRAS(G12V)-induced LC3 lipidation and puncta in both FHC cells expressing the KRAS(G12V) and Dox-induced KRAS(G12V) HEK293T cells, as well as autophagic turnover in the Dox-induced KRAS(G12V) HEK293T cells (Fig. 2g–q). In addition, adding exogenous PI4P reversed this effect in the Dox-induced KRAS(G12V) HEK293T cells (Fig. 2i–q). These data indicate that oncogenic KRAS induces RINCAA through a PI4P-dependent mechanism and PI4KB-mediated generation of PI4P can substitute for the canonical PI3K function during RINCAA.

### Inhibiting PI4KB decreases the proliferation of tumor cells with RAS mutations

KRAS-induced autophagic flux supports the survival of tumor cells.^70–73^ To determine the role of PI4KB in regulating RINCAA and cell proliferation in tumors, we treated two CRC cell lines with different KRAS mutations, such as HCT116 (KRAS^G13D^) and SW620 (KRAS^G12V^), with PIK93 or in combination with the MEK inhibitor trametinib. Consistent with the previous work,^74^ trametinib reduced the proliferation of HCT116 and SW620 cells in vitro. Additionally, we observed inhibited cell proliferation by PIK93 and a combined effect with trametinib (Supplementary information, Fig. S4a, b). We next used a subcutaneous xenograft tumor model and the two KRAS-mutated cell lines in NOD/SCID mice. Tumors were treated with the control, trametinib, PIK93, or a combination. The animals were followed to monitor tumor size. Consistent with the cellular data, trametinib and PIK93 treatment alone slowed tumor growth in KRAS-mutant tumors, and the drug combination further inhibited tumor growth (Supplementary information, Fig. S4c–h). Immunofluorescence and immunohistochemistry (IHC) analyses revealed decreased LC3 puncta and increased P62 in PIK93-treated mice and decreased p-ERK in tumors from trametinib-treated mice. The cell proliferation marker Ki67 was decreased in the single-treated group and further decreased in the double-treated group (Supplementary information, Fig. S4i–l). These data indicate that inhibiting PI4K reduced tumor cell autophagy and proliferation, and this strategy can be combined with MEK inhibition to enhance the tumor inhibitory effect, which is consistent with several studies showing the beneficial effect of inhibiting autophagy together with MEK inhibition when treating cancer.^18,39,40^

### WIPI2 acts as a PI4P effector in RINCAA

**Fig. 3:**
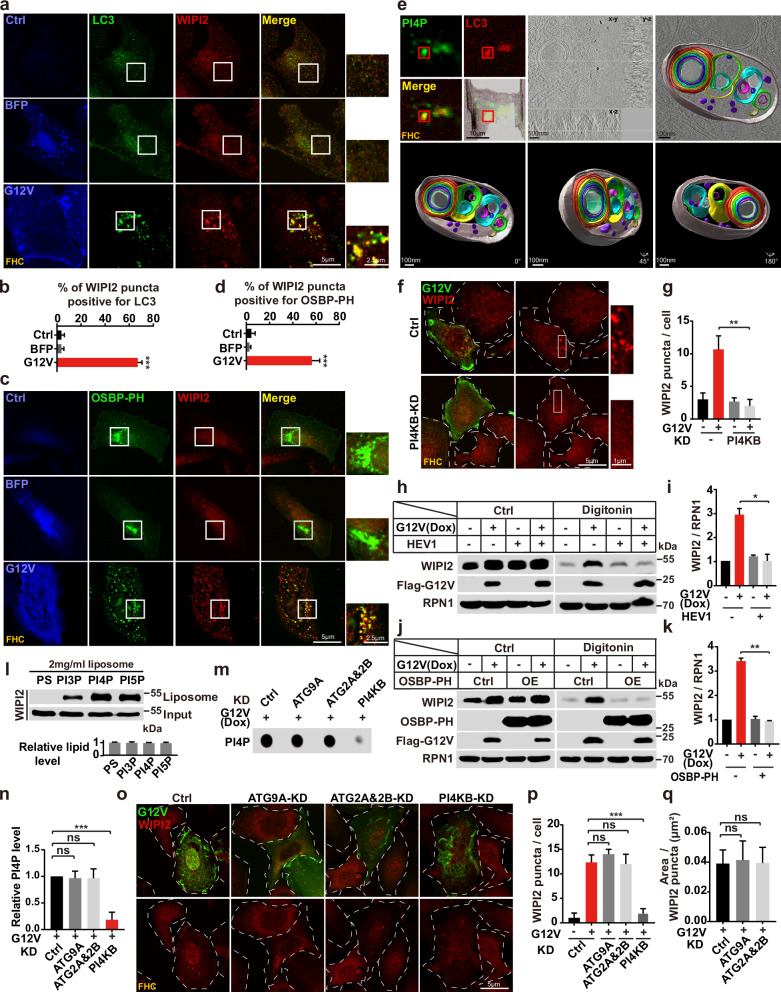
WIPI2 acts as a PI4P effector during RINCAA. **a** Immunofluorescence and confocal microscopy imaging were performed to visualize the colocalization between the WIPI2 puncta and the LC3 puncta in control and KRAS(G12V) cells. Representative cell images are shown. Scale bar sizes are indicated in the image. **b** Quantification of the percentage of the WIPI2 puncta colocalizing with the LC3 puncta in **a** (mean ± SEM). Three independent experiments (50 cells for each group/experiment) were performed for the statistical analysis (two-tailed *t*-test). ***P < 0.001. **c** Immunofluorescence and confocal microscopy imaging were performed to visualize the colocalization between the WIPI2 puncta and the OSBP-PH puncta in control and KRAS(G12V) cells. Representative cell images are shown. Scale bar sizes are indicated in the image. **d** Quantification of the percentage of the WIPI2 puncta colocalizing with the OSBP-PH puncta in **c** (mean ± SEM). Three independent experiments (50 cells for each group/experiment) were performed for the statistical analysis (two-tailed *t*-test). ***P < 0.001. **e** Light and electron microscopy combined with tomography images of the structure of LC3- and PI4P-positive compartments in KRAS(G12V) FHC cells. Scale bar sizes are indicated in the image. **f** Immunofluorescence analysis of the KRAS(G12V)-induced WIPI2 puncta with or without PI4KB knockdown. Representative cell images are shown. Scale bar sizes are indicated in the image. **g** Quantification of the number of the WIPI2 puncta in **f** (mean ± SEM). Three independent experiments (50 cells for each group/experiment) were performed for the statistical analysis (two-tailed *t*-test). **P < 0.01. **h** Immunoblot analysis of membrane-bound WIPI2 in control and KRAS(G12V) cells treated with the PI4KB inhibitor T-00127-HEV1 (10 μM). **i** Quantification of the levels of membrane-bound WIPI2 in **h** (mean ± SEM). Three independent experiments were performed for the statistical analysis (two-tailed *t*-test). *P < 0.05. **j** Immunoblot analysis of membrane-bound WIPI2 in control and KRAS(G12V) cells overexpressing OSBP-PH. **k** Quantification of the levels of membrane-bound WIPI2 in **j** (mean ± SEM). Three independent experiments were performed for the statistical analysis (two-tailed *t*-test). **P < 0.01. **l** The liposome flotation assay showing the binding of WIPI2 to PI3P, PI4P, and PI5P. The total lipid level of liposomes was normalized by PC. **m** Dot blot analysis of the KRAS(G12V)-induced PI4P generation in KRAS(G12V) HEK293T cells with or without knockdown of ATG9A, ATG2A&2B or PI4KB. **n** Quantification of the results in **m** (mean ± SEM). Three independent experiments were performed for the statistical analysis (two-tailed *t*-test). ***P < 0.001, ns no significance. **o** Immunofluorescence analysis of the KRAS(G12V)-induced WIPI2 puncta with knockdown of ATG9A, ATG2A&2B or PI4KB in FHC cells. Representative cell images are shown. Scale bar sizes are indicated in the image. **p** Quantification of the number of the WIPI2 puncta in **o** (mean ± SEM). Three independent experiments (50 cells for each group/experiment) were performed for the statistical analysis (two-tailed *t*-test). ***P < 0.001, ns no significance. **q** Quantification of areas of the WIPI2 puncta in **o** (mean ± SEM). Three independent experiments (50 cells for each group/experiment) were performed for the statistical analysis (two-tailed *t*-test), ns no significance.

The mammalian orthologue of yeast Atg18, WIPI2, is a WD40-repeat-containing PI3P-binding protein initially reported to facilitate LC3 lipidation and the subsequent growth of the phagophore by recruiting the ATG12–ATG5/ATG16L1 complex.^75^ In addition to binding PI3P, WIPI2 interacts with PI4P and PI5P.^76,77^ Because our data indicate that PI4KB, PI4P, and WIPI2 are required for RINCAA, we sought to test whether WIPI2 is the PI4P effector. WIPI2 formed puncta, which colocalized with LC3 upon KRAS(G12V) induction, indicating that WIPI2 localizes on the RIMMBA during RINCAA (Fig. 3a, b). The WIPI2 puncta also overlapped with the PH domain of OSBP (OSBP-PH which binds to PI4P) with an optimal expression level, suggesting a link between PI4P, WIPI2, and autophagic membranes (Fig. 3c, d).

The data identified RIMMBA as alternative autophagosomes in RAS-induced autophagy. Using cryo-correlative light and electron microscopy (CLEM) combined with electron tomography (ET), we characterized the structure of LC3- and PI4P-positive compartments. ET consistently demonstrated an autophagosome-sized structure with a limiting membrane enclosing multivesicular and multilaminar vesicles (Fig. 3e; Supplementary information, Video S1). Therefore, these findings establish a connection between RIMMBA and the presence of PI4P and LC3, suggesting that RIMMBA is an early structure of RINCAA equivalent to the double-membrane autophagosome in canonical autophagy.

We performed the following experiments to determine whether the location of WIPI2 on RIMMBA was dependent on PI4P. In one experiment, we knocked down PI4KB, which decreased the number of WIPI2 puncta induced by KRAS(G12V) (Fig. 3f, g). In the other two experiments, we reduced PI4P via T-00127-HEV1 treatment or limited the accessibility of PI4P by overexpressing OSBP-PH. In both conditions, decreasing the level or accessibility of PI4P reduced the amount of membrane-bound WIPI2 as revealed by a membrane isolation assay (Fig. 3h–k). Taken together, these data indicate that PI4P is required for WIPI2 targeting to the autophagic membrane.

To confirm WIPI2 binding, we analyzed the binding capacity between WIPI2 and PI4P using a lipid flotation assay. The results showed that WIPI2 bound to PI4P with a higher capacity than WIPI2 to PI3P or PI5P (Fig. 3l). In line with the above data of RINCAA analysis, ATG9A and ATG2s were not required for PI4P production and WIPI2 puncta formation with KRAS(G12V) (Fig. 3m–q). ATG9A and ATG2s were not involved in regulating PI4P levels in multiple cancer cell lines with RAS mutations (Supplementary information, Fig. S6o). Collectively, these data indicate that WIPI2 acts as a PI4P effector in RINCAA.

### The P38-ULK1-PI4KB-WIPI2 cascade regulates RINCAA

**Fig. 4:**
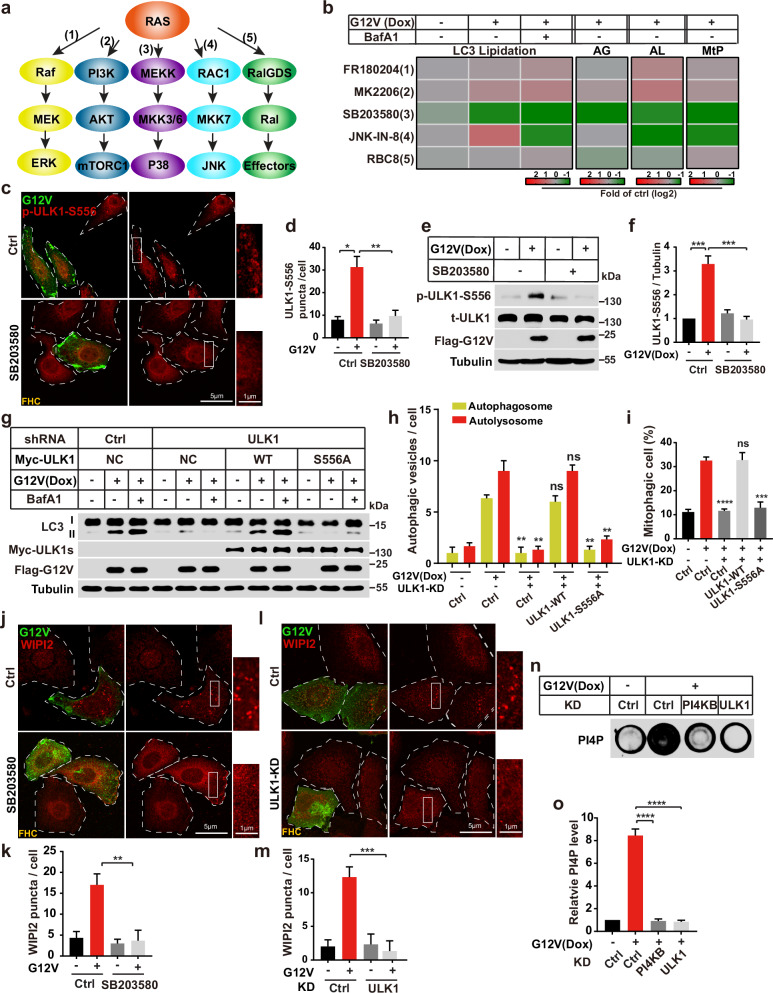
The P38-ULK1-PI4K-WIPI2 cascade regulates RINCAA. **a** The schematic diagram of the RAS signaling pathway. **b** Heatmap showing the changes of LC3 lipidation (related to Supplementary information, Fig. S5b, c), autophagic flux (AG and AL, related to Supplementary information, Fig. S5d, e), and mitophagy (MtP, related to Supplementary information, Fig. S5f, g) in control and KRAS(G12V) HEK293T cells with different inhibitors in the RAS signaling pathway. Color represents the log_2_ (fold) of counts per samples. **c** Immunofluorescence analysis of the regulation of SB203580 on the KRAS(G12V)-induced p-ULK1-S556 puncta. Representative cell images are shown. Scale bar sizes are indicated in the image. **d** Quantification of the numbers of p-ULK1-S556 puncta in **c** (mean ± SEM). Three independent experiments (50 cells for each group/experiment) were performed for the statistical analysis (two-tailed *t*-test). *P < 0.05; **P < 0.01. **e** Immunoblot analysis of the regulation of SB203580 on the KRAS(G12V)-induced p-ULK1-S556 level. **f** Quantification of the results in **e** (mean ± SEM). Three independent experiments were performed for the statistical analysis (two-tailed *t*-test). ***P < 0.001. **g** Immunoblot analysis of the rescue effects of WT-ULK1 and ULK1-S556A on the KRAS(G12V)-induced LC3 lipidation when knocking down ULK1. **h** Quantification of the yellow (RFP^+^GFP^+^) and Red (RFP^+^GFP^−^) LC3 puncta (related to Supplementary information, Fig. S5h) to analyze the rescue effects of WT-ULK1 and ULK1(S556A) on the KRAS(G12V)-induced LC3 flux in HEK293T cells. Data are presented as mean ± SEM. Three independent experiments (50 cells for each group/experiment) were performed for the statistical analysis (two-tailed *t*-test). **P < 0.01, ns no significance. **i** Quantification of results shown in Supplementary information, Fig. S5i. The percentage of cells with mitophagy was based on Y610-mCherry/V610. Data are presented as mean ± SEM. Three independent experiments were performed for the statistical analysis (two-tailed *t*-test). ***P < 0.001; ****P < 0.0001, ns no significance. **j** Immunofluorescence analysis of the regulation of SB203580 on the KRAS(G12V)-induced WIPI2 puncta in FHC cells. Representative cell images are shown. Scale bar sizes are indicated in the image. **k** Quantification of the numbers of WIPI2 puncta in **j** (mean ± SEM). Three independent experiments (50 cells for each group/experiment) were performed for the statistical analysis (two-tailed *t*-test). **P < 0.01. **l** Immunofluorescence analysis of the KRAS(G12V)-induced WIPI2 puncta with ULK1 knockdown in FHC cells. Representative cell images are shown. Scale bar sizes are indicated in the image. **m** Quantification of the numbers of WIPI2 puncta in **l** (mean ± SEM). Three independent experiments (50 cells for each group/experiment) were performed for the statistical analysis (two-tailed *t*-test). ***P < 0.001. **n** Dot blot analysis of the KRAS(G12V)-induced PI4P generation with PI4KB or ULK1 knockdown in HEK293T cells. **o** Quantification result for **n** (mean ± SEM). Three experiments were performed for the statistical analysis (two-tailed *t*-test). ****P < 0.0001.

RAS activates the ERK, PI3K/AKT, P38, JNK, and Ral pathways, resulting in distinct cellular responses (Fig. 4a).^10,78–80^ To examine which of these downstream pathways is involved in RINCAA, we tested a series of specific inhibitors. The effects of the inhibitors were validated by their targets (Supplementary information, Fig. S5a). The P38-specific inhibitor SB203580^81^ reduced LC3 lipidation in Dox-induced cells compared to the control, whereas inhibitors of ERK (FR180204),^82^ PI3K/AKT (MK2206),^83^ and Ral (RBC8)^84^ did not (Fig. 4b; Supplementary information, Fig. S5b, c). The effect of inhibiting P38 on RINCAA was further confirmed by the autophagic turnover assays including the double-fluorescence LC3 assay and the mito-keima assay (Fig. 4b; Supplementary information, Fig. S5d–g). The JNK inhibitor JNK-IN-8^85^ increased LC3 lipidation upon KRAS(G12V) expression but reduced LC3 lipidation after treatment with Baf A1, suggesting an effect on the maturation of the RIMMBA. This notion was confirmed by autophagic turnover assays, in which inhibiting JNK did not affect the yellow puncta but decreased the red puncta in the double-fluorescence LC3 assay (Supplementary information, Fig. S5d, e). JNK inhibition also decreased mitophagy in the mito-keima assay (Supplementary information, Fig. S5f, g).

Because inhibiting P38 blocks the biogenesis of RIMMBA, we next focused on this pathway. The P38-MAPK pathway mediates the activation of autophagy by activating mouse ULK1 through phosphorylation on serine (S) 555.^86^ We observed the formation of ULK1-S556 puncta (an indicator of ULK1 phosphorylation and early autophagosome formation, human ULK1 S556 corresponds to mouse ULK1 S555) upon KRAS(G12V) expression, which was mitigated by P38 inhibition (Fig. 4c, d). Immunoblot analysis confirmed the increase in ULK1 S556 phosphorylation, which was inhibited by SB203580 (Fig. 4e, f). We next performed rescue experiments in ULK1-depleted cells. Restoration of wild-type (WT) ULK1 but not of a ULK1(S556A) point mutant restored LC3 lipidation upon KRAS(G12V) expression (Fig. 4g–i; Supplementary information, Fig. S5h, i), indicating that P38-mediated activation of ULK1 on S556 is important for RINCAA. The requirement of P38 activation in RINCAA was also confirmed in multiple cancer cell lines (HCT116, SW620, H1299, A549, MiaPaCa2, and SW1990) with endogenous RAS mutation (Supplementary information, Fig. S5j–l). Of note, another study also highlighted the significance of ULK1 S556 phosphorylation in activating autophagy in RAS-mutant cancer cell lines. This study demonstrated that an MEK inhibitor enhances LKB1 and AMPK1 activation, subsequently leading to ULK1 S556 phosphorylation and the enhancement of autophagy.^40^

Next, we determined the relationship between P38, ULK1, PI4P, and WIPI2. SB203580 treatment or ULK1 knockdown inhibited the formation of WIPI2 puncta induced by KRAS(G12V) expression (Fig. 4j–m). ULK1 knockdown blocked the increase in PI4P induced by KRAS(G12V) expression similar to PI4KB depletion (Fig. 4n, o). These data indicate that P38 activates ULK1 via S556 phosphorylation and ULK1 activates PI4P biogenesis and WIPI2 targeting to early RIMMBA structures in the context of oncogenic KRAS signaling.

### ULK1-mediated PI4KB phosphorylation is required for RINCAA and KRAS-mutated tumor growth

**Fig. 5:**
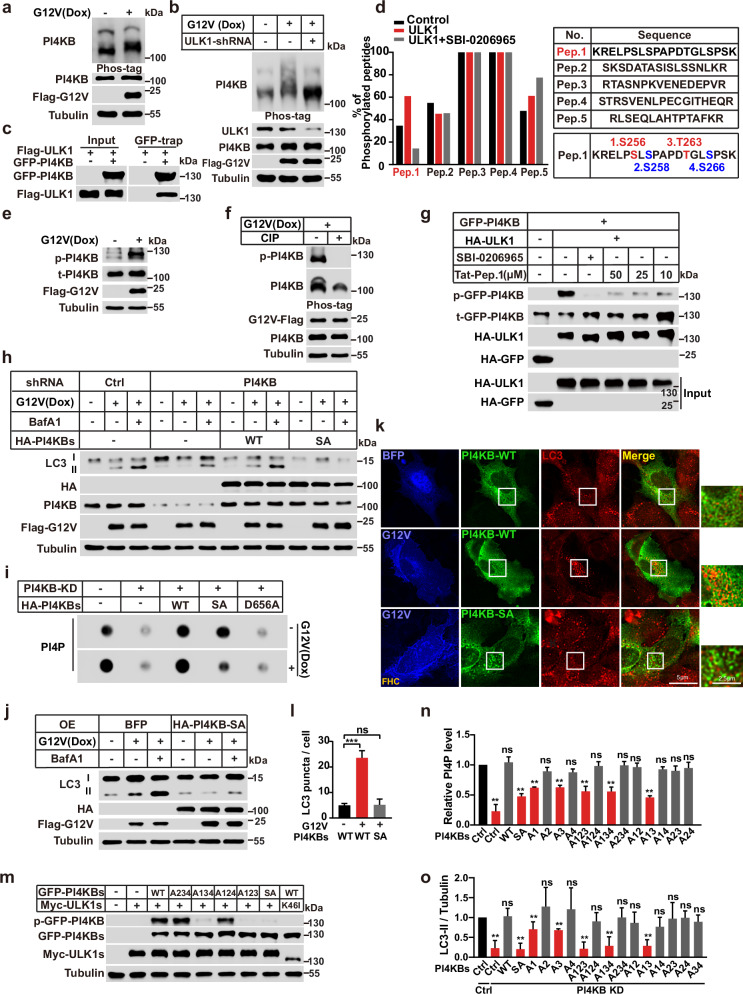
ULK1 mediates PI4KB phosphorylation on the region of K251–K269 which is required for RINCAA. **a** Immunoblot analysis of the PI4KB phosphorylation in control and KRAS(G12V) HEK293T cells by phos-tag gel. **b** Immunoblot analysis of the PI4KB phosphorylation in control and KRAS(G12V) HEK293T cells with or without ULK1 knockdown by phos-tag gel. **c** Co-IP analysis for the interaction between PI4KB and ULK1. **d** MS analysis of the percentage of the five phosphorylated PI4KB peptides. **e** Immunoblot analysis of the PI4KB phosphorylation by the antibody against p-PI4KB in control and KRAS(G12V) HEK293T cells. **f** Immunoblot analysis of the PI4KB phosphorylation by the antibody against p-PI4KB in KRAS(G12V) HEK293T cells treated with or without CIP. **g** Immunoprecipitation and in vitro kinase assay to analyze the PI4KB phosphorylation by ULK1. HEK293T cells were transfected with HA-tagged ULK1 constructs, lysed, and used for immunoprecipitation reactions. Immunoprecipitates were incubated in an in vitro kinase reaction mixture containing ATP and the substrate GFP-PI4KB with or without SBI-0206965 (10 μM) and Peptide-1(Tat-Pep.1) at indicated concentrations. Reaction products were resolved on SDS-PAGE gels. Anti-HA immunoblotting was performed as a control to quantify the amount of ULK1 protein precipitated. The PI4KB phosphorylation was detected by the antibody against p-PI4KB. The experiment was repeated three times. **h** Immunoblot analysis of the rescue effect of PI4KB-WT or PI4KB-SA on KRAS(G12V)-induced LC3 lipidation after knocking down PI4KB in HEK293T cells in the absence or presence of 500 nM Baf A1 for 1.5 h. **i** Dot blot analysis of the effect of PI4KB mutants on the KRAS(G12V)-induced PI4P generation in HEK293T cells. **j** Immunoblot analysis of the KRAS(G12V)-induced LC3 lipidation when overexpressing PI4KB-SA in HEK293T cells in the absence or presence of 500 nM Baf A1 for 1.5 h. **k** Immunofluorescence analysis of the KRAS(G12V)-induced LC3 puncta when overexpressing PI4KB-SA in FHC cells. Representative cell images are shown. Scale bar sizes are indicated in the image. **l** Quantification of the LC3 puncta in **k** (mean ± SEM). Three independent experiments (50 cells for each group/experiment) were performed for the statistical analysis (two-tailed *t*-test). ***P < 0.001, ns no significance. **m** Immunoblot analysis of changes of the p-PI4KB by the different site mutations of PI4KB (S256(1), S258(2), T263(3) and S266(4)), A234 (S258A, T263A, S266A), A134 (S256A, T263A, S266A), A124 (S256A, S258A, S266A), A123(S256A, S258A, T263A) or SA in control and ULK1-overexpressing HEK293T cells. **n** Quantification of PI4P level by the different site mutation of PI4KB-Peptide-1 in KRAS(G12V) HEK293T cells in experiments shown by Supplementary information, Fig. S6d (mean ± SEM). Three independent experiments were performed for the statistical analysis (two-tailed *t*-test). **P < 0.01, ns no significance. **o** Statistical analysis of LC3 lipidation by overexpressing different site mutations of PI4KB (S256(1), S258(2), T263(3) and S266(4)) in control and PI4KB-knockdown KRAS(G12V) HEK293T cells in experiments shown by Supplementary information, Fig. S6e (mean ± SEM). Three independent experiments were performed for the statistical analysis (two-tailed *t*-test). **P < 0.01, ns no significance.

To understand how activating ULK1 affects PI4KB, we conducted a phos-tag gel assay, in which KRAS(G12V) overexpression caused a gel mobility shift of PI4KB to a higher molecular weight, suggesting more phosphorylation (Fig. 5a). Interestingly, ULK1 knockdown reversed the mobility shift of PI4KB upon KRAS(G12V) expression (Fig. 5b). In addition, ULK1 was associated with PI4KB in a co-immunoprecipitation (co-IP) experiment (Fig. 5c). Therefore, these data indicate that ULK1 is involved in PI4KB phosphorylation. Mass spectrometry (MS) analysis identified five PI4KB phosphorylated peptides in which phosphorylation of Peptide-1 (K251–K269) indicated regulation by ULK1 activity (Fig. 5d; Supplementary information, Table S1). This peptide contains S256, S258, T263, and S266 as potential ULK1 phosphorylation sites.

To further elucidate PI4KB phosphorylation at this region, we developed a phosphorylation-specific antibody targeting its four potential phosphorylation sites. Upon induction of KRAS(G12V) expression, we observed an augmented phosphorylation signal, which was abrogated by treatment with calf-intestinal alkaline phosphatase, confirming the antibody’s specificity (Fig. 5e, f). The antibody’s specificity was further validated in HCT116, SW620 and MiaPaCa2 cells, where the phosphorylation signal in immune blot or immune staining was inhibited by RAS knockdown or AMG510, respectively (Supplementary information, Fig. S6a–c). Utilizing this antibody, we investigated whether ULK1 can directly phosphorylate S256, S258, T263, and S266 of PI4KB. Immune-isolated ULK1 and PI4KB were subjected to an in vitro reaction with ATP. Our results indicate that the phosphorylation occurs in the presence of ULK1, an effect that was fully abrogated by treatment with the ULK1 inhibitor SBI-0206965. Additionally, synthesized Peptide-1 (K251–K269 of PI4KB), likely competing with PI4KB for ULK1 phosphorylation, also abolished the phosphorylation (Fig. 5g).

To determine the function of PI4KB phosphorylation on region K251–K269, we generated a phosphorylation-deficient mutant by mutating the four potential phosphorylation sites to alanine (A) (SA mutant). Re-expression of WT PI4KB in PI4KB-knockdown cells restored LC3 lipidation upon KRAS(G12V) expression, whereas the SA mutant did not (Fig. 5h), suggesting that phosphorylation of region K251–K269 is required for PI4KB to regulate RINCAA. To analyze the effect of the SA mutation on kinase activity, we performed a PI4P dot blot assay. Knockdown of PI4KB in control cells decreased PI4P production, which was restored by the WT and SA PI4KB but not by the kinase-dead mutant (PI4KB (D656A)) (Fig. 5i). However, WT PI4KB but not the SA mutant restored PI4P production upon PI4KB depletion in KRAS(G12V)-expressing cells. Taken together, these data suggest that the phosphorylation of region K251–K269 may specifically regulate RINCAA.

**Fig. 6:**
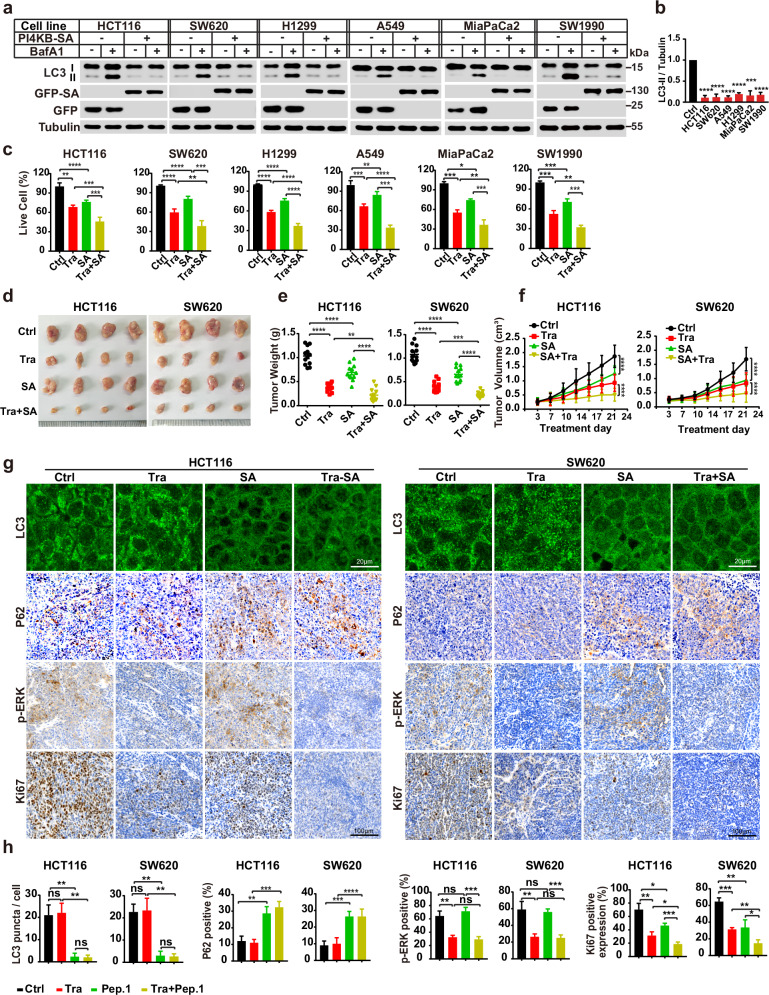
Inhibitory effect of PI4KB-SA on RAS-mutant cell proliferation and xenograft tumor. **a** Immunoblot analysis of the inhibitory effect of the PI4KB-SA on LC3 lipidation in the control and RAS-mutant cancer cell lines in the absence or presence of 500 nM Baf A1 for 1.5 h. **b** Quantification of the ratio of lipidated LC3 to tubulin with the control set as 1.00 (control cells with Baf A1) analyzed in **a** (mean ± SEM). Three independent experiments were performed for the statistical analysis (two-tailed *t*-test). ***P < 0.01; ****P < 0.0001. **c** The CCK8 analysis of the cell proliferation of the control and RAS-mutant cell lines treated with the PI4KB-SA overexpression (SA) combined with Trametinib (Tra, 100 nM) for 96 h (mean ± SEM). Three independent experiments were performed for the statistical analysis (two-tailed *t*-test). *P < 0.05; **P < 0.01; ***P < 0.001; ****P < 0.0001. **d** Images of xenograft tumors of HCT116 and SW620 cells from mice treated with: (1) vehicle (Ctrl); (2) Trametinib (Tra); (3) PI4KB-SA overexpression (SA); or (4) the combination of both (Tra + SA) (*n* = 12 mice (one tumor/mice) in each group). The tumors were removed and photographed after 24-day treatment. **e** Weights of the xenograft tumors in **d** (means ± SD). The statistical analysis was performed by two-tailed *t*-test. **P < 0.01; ***P < 0.001, ****P < 0.0001. **f** The growth curve of the xenograft tumors in **d** (means ± SD). Statistical analysis was performed by two-way-ANOVA; ****P < 0.0001. **g** Immunofluorescence and IHC analyses of the sections of xenograft tumors in **d**. Sections were stained with antibody against LC3, P62, p-ERK1/2 or Ki67, as indicated. Scale bar sizes are indicated in the image. **h** Statistical analysis of the numbers of LC3 puncta, P62 positive rates, p-ERK positive rates, and levels of Ki67 expression in **g** (means ± SD). Statistical analysis was performed by two-tailed *t*-test; *P < 0.05; **P < 0.01; ***P < 0.001, ns no significance.

In addition to loss of function, the PI4KB SA mutant generated a dominant-negative effect on RINCAA when overexpressed, as revealed by LC3 lipidation and puncta formation (Fig. 5j–l). Consistently, overexpressing PI4KB-SA inhibited RINCAA and cell proliferation in the RAS-mutant cell lines (HCT116, SW620, H1299, A549, MiaPaCa2, and SW1990) (Fig. 6a–c). Therefore, we subsequently determined the anti-neoplastic effects of PI4KB-SA expression using the subcutaneous xenograft tumor model. We generated HCT116 and SW620 cell lines stably overexpressing PI4KB-SA which were employed in the xenograft tumor experiment. Consistent with inhibiting PI4KB, PI4KB-SA expression reduced RINCAA (as revealed by LC3 and P62 staining), cell proliferation (Ki67), and tumor growth. The tumor inhibitory effect was enhanced by combining with the MEK inhibitor treatment (Fig. 6d–h). These findings indicate that ULK1-mediated PI4KB phosphorylation is required for RINCAA and tumor growth.

The MS analysis shown above did not provide precise identification of the phosphorylation sites on Peptide 1. However, through experimentation involving various combinations of phosphorylation site mutations, we discovered that the simultaneous mutation of sites 1 and 3 (S256 and T263) instead of the other two possible sites resulted in the abolition of PI4KB phosphorylation (Fig. 5m). Such mutation also impaired the increase of PI4P mediated by PI4KB and compromised RINCAA in the presence of KRAS(G12V) expression (Fig. 5n, o; Supplementary information, Fig. S6d, e). These findings suggest that S256 and T263 are the primary sites of PI4KB phosphorylation crucial for RINCAA activation. A preferred amino acid sequence for ULK1 substrates, characterized by a Leu or Met residue at position −3, along with aliphatic and aromatic hydrophobic residues at positions +1 and +2, has been identified.^87^ The neighboring residues of S256 and T263 contains a Leu at position +1 and +2 respectively and therefore partially match this sequence.

### ULK1-regulated PI4KB phosphorylation may be a target for treating RAS-mutant cancer

**Fig. 7:**
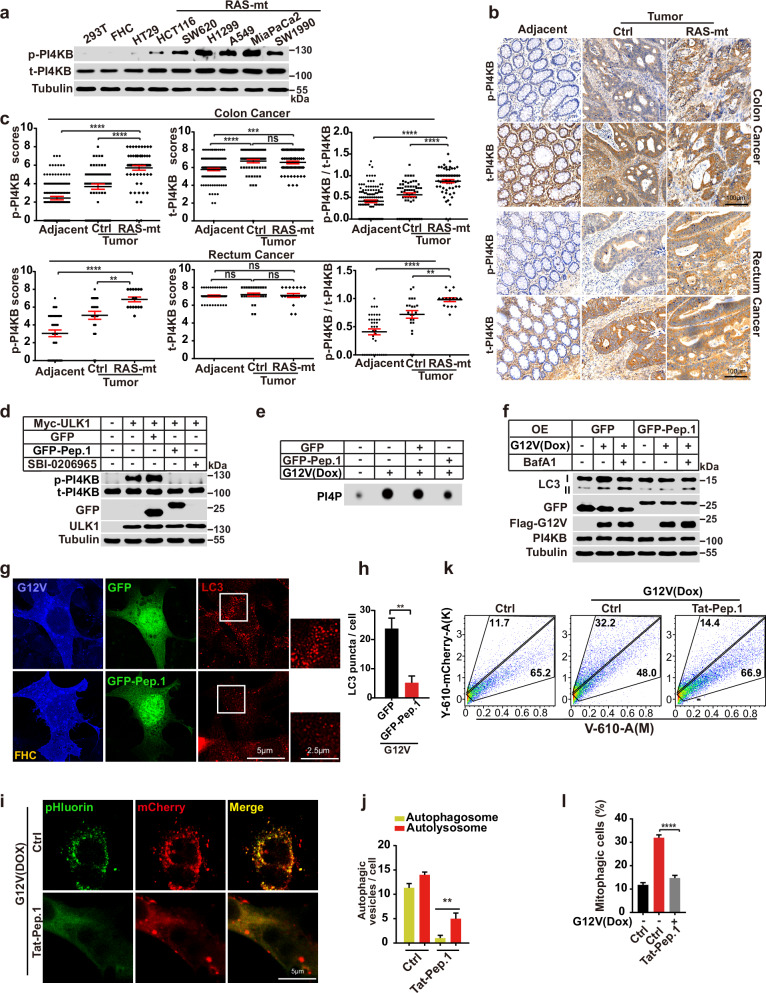
Targeting ULK1-mediated PI4KB phosphorylation on S256 and T263 could inhibit RINCAA. **a** Immunoblot analysis of the level of p-PI4KB in different cancer cell lines. **b** Representative IHC staining of p-PI4KB of the tissue microarrays containing CRC tissues (control and RAS-mutant) and adjacent normal tissues. **c** Quantification of the p-PI4KB level of the tissue microarrays with colon cancer (Adjacent: *n* = 119; Cancer: *n* = 60 (Ctrl) and *n* = 59 (RAS-mutant)) and rectum cancer (Adjacent: *n* = 37; Cancer: *n* = 22 (Ctrl) and *n* = 15 (RAS-mutant)) (means ± SDs) patient samples. The statistical analysis was performed by the Wilcoxon signed rank test. **P < 0.01; ***P < 0.001; ****P < 0.0001, ns no significance. **d** Immunoblot analysis of the PI4KB phosphorylation by the antibody against p-PI4KB in HEK293T cells expressing Myc-ULK1 with or without GFP-Peptide-1 (GFP-Pep.1) overexpression or SBI-0206965 (10 μM) treatment. **e** Dot blot analysis of the effect of GFP-Peptide-1 on the KRAS(G12V)-induced PI4P generation. **f** Immunoblot analysis of the KRAS(G12V)-induced LC3 lipidation when overexpressing GFP-Peptide-1 in the absence or presence of 500 nM Baf A1 for 1.5 h. **g** Immunofluorescence analysis of the KRAS(G12V)-induced LC3 puncta with or without overexpressing GFP-Peptide-1. Representative images are shown. Scale bar sizes are indicated in the image. **h** Quantification of the LC3 puncta in **g** (mean ± SEM). Three experiments (50 cells for each group/experiment) were performed for the statistics (two-tailed *t-*test). **P < 0.01. **i** Detection of effect of GFP-Peptide-1 on autophagy flux by the tandem fluorescent LC3 system in KRAS(G12V) cells stably expressing mCherry-pHluorin-LC3B. Representative cell images are shown. Scale bar sizes are indicated in the image. **j** Quantification of the yellow (RFP^+^GFP^+^) and Red (RFP^+^GFP^−^) LC3 puncta. Data are presented as mean ± SEM. Three experiments (50 cells for each group/experiment) were performed for the statistics (two-tailed *t*-test). **P < 0.01. **k** FACS analysis of control and KRAS(G12V) cells co-expressing mt-Keima and Parkin with or without GFP-Peptide-1 expression using V610 and Y610-mCherry detectors (Beckman CytoFLEX LX). The FACS results are representative of at least three independent experiments. **l** The percentage of cells with mitophagy based on Y610-mCherry/V610 calculated for **k**. Data are represented as mean ± SEM. Three experiments were performed for the statistics (two-tailed *t*-test). ****P < 0.0001.

The data indicate that ULK1-regulated PI4KB phosphorylation of S256 and T263 may be a signature of KRAS overactivation and the induction of RINCAA. In immunoblot analysis, cancer cells with a RAS mutation contained a higher level of PI4KB phosphorylation on S256 and T263 than those without a RAS mutation (Fig. 7a). In addition, in a mutant-RAS tissue microarray of patients with CRC, the overall PI4KB-Peptide 1 phosphorylation was much higher in RAS-mutated (compared to those with WT RAS) colon or rectal cancer (Fig. 7b, c). The data suggest that upregulated PI4KB-Peptide 1 phosphorylation may be a signature of cancer with the RAS mutation.

We investigated the impact of phosphorylation substrate PI4KB-Peptide-1 on ULK1-regulated PI4KB phosphorylation. Consistent with our in vitro ULK1 kinase assay (Fig. 5g), expression of PI4KB-Peptide-1 or treating cell with PI4KB-Peptide-1 fused to Tat led to the inhibition of PI4KB phosphorylation on S256 and T263, resulting in decreased PI4P generation and RINCAA blockage as demonstrated by multiple assays, including LC3 lipidation, tandem fluorescence LC3 assay and mitophagy (Fig. 7d–l). Therefore, the data confirm the notion that competing against PI4KB phosphorylation on S256 and T263 blocks RINCAA.

**Fig. 8:**
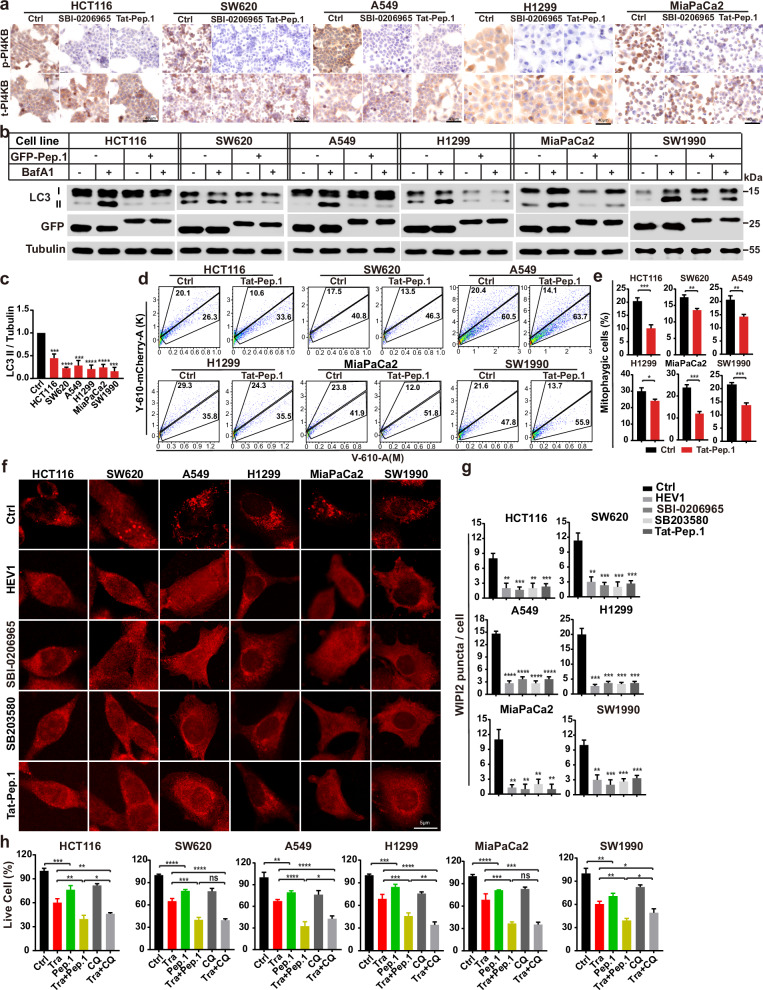
The PI4KB-Peptide-1 inhibits RAS-mutant cell autophagy and growth. **a** IHC analysis of HCT116, SW620, A549, H1299 and MiaPaCa-2 cells treated with or without SBI-0206965 (10 μM) or Peptide-1 (Tat-Pep.1, 25 μM) for 2 h. Cells were stained with antibody against PI4KB and p-PI4KB as indicated. Scale bar sizes are indicated in the image. **b** Immunoblot analysis of LC3 lipidation of HCT116, SW620, A549, H1299 and MiaPaCa-2 cells with or without overexpression of GFP-Peptide-1 (GFP-Pep.1) in the absence or presence of 500 nM Baf A1 for 1.5 h. **c** Quantification of the ratio of lipidated LC3 to tubulin with the control set as 1.00 (control cells with Baf A1) analyzed in **b** (mean ± SEM). Three independent experiments were performed for the statistic analysis (two-tailed *t*-test). ***P < 0.01; ****P < 0.0001. **d** FACS analysis of HCT116, SW620, A549, H1299 and MiaPaCa-2 cells co-expressing mt-Keima and Parkin with or without GFP-Peptide-1 expression using V610 and Y610-mCherry detectors (Beckman CytoFLEX LX). The FACS results are representative of at least three independent experiments. **e** The percentage of cells with mitophagy based on Y610-mCherry/V610 calculated for **d**. Data are represented as mean ± SEM. Three experiments were performed for the statistics (two-tailed *t*-test). *P < 0.05; **P < 0.01; ***P < 0.001. **f** Immunofluorescence analysis of the WIPI2 puncta of different cancer cell lines treated with HEV1 (10 μM), SBI-209626 (10 μM), SB203580 (10 μM) for 1.5 h or overexpressing GFP-Peptide-1. Representative cell images are shown. Scale bar sizes are indicated in the image. **g** Quantification of the WIPI2 puncta in **g**. Data are represented as mean ± SEM. Three independent experiments (50 cells for each group/experiment) were performed for the statistics (two-tailed *t*-test). **P < 0.01; ***P < 0.01; ****P < 0.0001. **h** The CCK8 analysis of the cell proliferation of HCT116, SW620, A549, H1299 and MiaPaCa-2 cells treated with vehicle (Ctrl), Trametinib (Tra, 100 nM), Tat-Peptide-1 (Pep.1, 5 μM), the combination of Trametinib and Tat-Peptide-1 (Tra + Pep.1), chloroquine (CQ, 10 μM), the combination of Trametinib and chloroquine (Tra + CQ) for 96 h (mean ± SEM). Three independent experiments were performed for the statistical analysis (two-tailed *t*-test). *P < 0.05; **P < 0.01; ***P < 0.001; ****P < 0.0001, ns no significance.

We then sought to confirm a similar inhibition of RINCAA by PI4KB phosphorylation on S256 and T263 using cancer cells with endogenous RAS mutations. Consistently, PI4KB-Peptide-1 mediated inhibition of PI4KB phosphorylation on S256 and T263 (Fig. 8a), and therefore hindered RINCAA, including LC3 lipidation (Fig. 8b, c), autophagic flux determined by mitophagy (Fig. 8d, e), and WIPI2 puncta formation, across various RAS-mutated cancer cell lines (Fig. 8f, g). Notably, PI4KB-Peptide-1 also suppressed cell proliferation in these cancer cells and exhibited comparable inhibitory effects on proliferation to chloroquine, a clinically used lysosomal inhibitor for autophagy inhibition, when combined with trametinib (Fig. 8h).

**Fig. 9:**
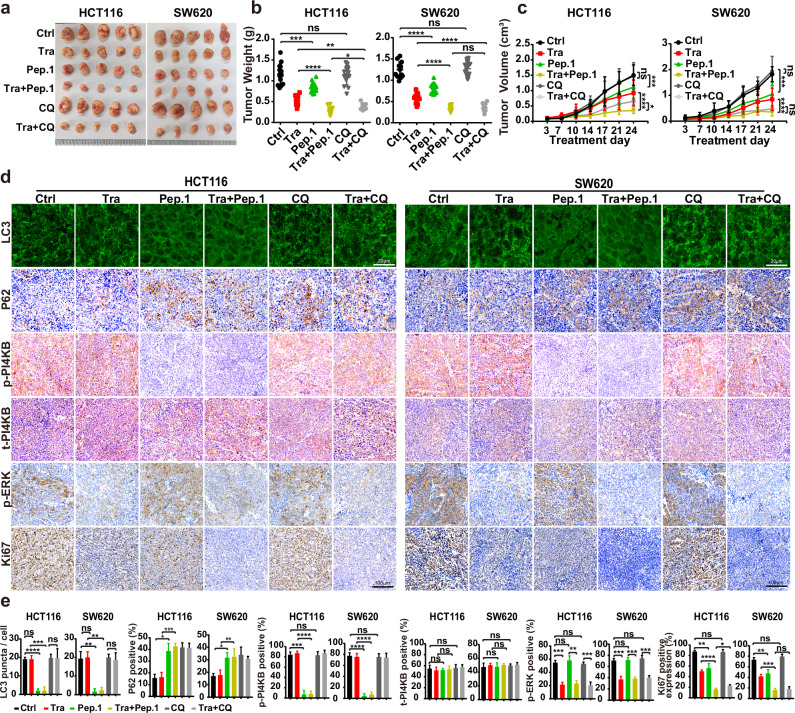
ULK1-mediated PI4KB phosphorylation on S256 and T263 may be a target for treating RAS-mutant cancer. **a** Images of xenograft tumors of HCT116 and SW620 cells from mice treated with: (1) vehicle (Control); (2) Trametinib (Tra); (3) GFP-Peptide-1 (GFP-Pep.1); or (4) the combination of Trametinib and GFP-Peptide-1 (Tra + Pep.1) (5) chloroquine (CQ); (6) the combination of Trametinib and chloroquine (Tra + CQ). *n* = 12 mice, one tumor/mice in each group. **b** Weights of the xenograft tumors in **a** (means ± SD). The statistical analysis was performed by two-tailed *t*-test. *P < 0.05; **P < 0.01; ***P < 0.001; ****P < 0.0001, ns no significance. **c** The growth curve of the xenograft tumors in **a** (means ± SD). Statistical analysis was performed by two-way ANOVA; *P < 0.05; ***P < 0.001; ****P < 0.0001, ns no significance. **d** Immunofluorescence and IHC analyses of sections of the xenograft tumors in **a**. Sections were stained with antibody against LC3, P62, p-PI4KB, PI4KB, p-ERK1/2 or Ki67, as indicated. Scale bars are located at the bottom right of the images. **e** Statistical analysis of the numbers of LC3 puncta, P62 positive rates, p-PI4KB positive rates, t-PI4KB positive rates, p-ERK positive rates, and levels of Ki67 expression in **d** (means ± SD). Statistical analysis was performed by two-tailed *t*-test. *P < 0.05; **P < 0.01; ***< 0.001; ****P < 0.0001, ns no significance.

We employed the tumor xenograft assay mentioned above to determine an inhibitory effect of tumor formation by blocking PI4KB phosphorylation on S256 and T263. In this in vitro tumor model, PI4KB-Peptide-1 expression in combination with trametinib dramatically blocked tumor growth, achieving equivalent (SW620) or superior (HCT116) tumor inhibitory effects to chloroquine combined with trametinib (Fig. 9a–c). Immunofluorescence and IHC assays confirmed that PI4KB-Peptide-1 expression curbed PI4KB phosphorylation on S256 and T263, RINCAA, cell proliferation, and tumor growth using xenograft model (Fig. 9d, e). Therefore, targeting PI4KB phosphorylation on S256 and T263 specifically inhibits RINCAA in RAS-mutant tumors and suppresses tumor growth.

**Fig. 10:**
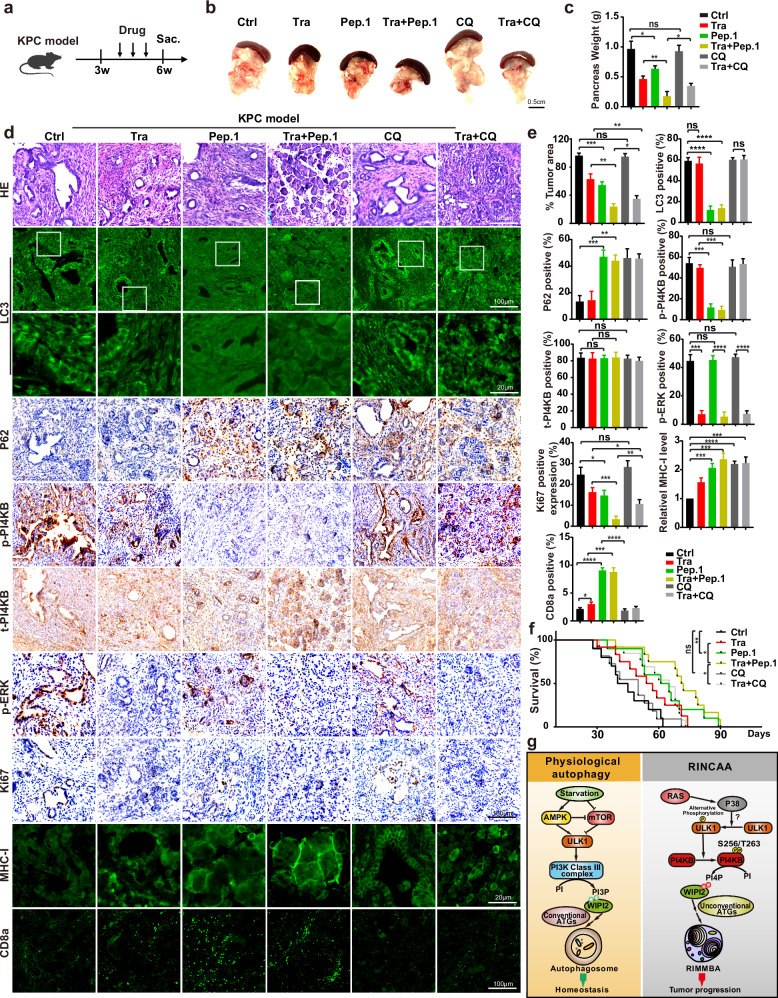
Inhibition of tumors by PI4KB-Peptide-1 in a KPC mouse model of pancreatic cancer. **a** Schematic diagram of experimental design in KPC mice. **b** Representative images from primary pancreatic tumors at endpoint analysis in indicated treatment groups: vehicle (Ctrl); Trametinib (Tra); Tat-Peptide-1 (Pep.1); the combination of Trametinib and Tat-Peptide-1 (Tra + Pep.1); chloroquine (CQ); the combination of Trametinib and chloroquine (Tra + CQ). **c** Weights of primary pancreatic tumors in **b** (means ± SD) (*n* = 10 mice). The statistical analysis was performed by two-tailed *t*-test. *P < 0.05; **P < 0.01. **d** Immunofluorescence and IHC analyses of sections of primary pancreatic tumors in **b**. H&E staining is provided to show the state of the tissue in each condition. Sections were stained with antibody against LC3, P62, p-PI4KB, PI4KB, p-ERK1/2, Ki67, MHC-I or CD8a as indicated. Scale bars are located at the bottom right of the images. **e** Statistical analysis of tumor area and the numbers of LC3 puncta, P62 positive rates, p-PI4KB positive rates, t-PI4KB positive rates, p-ERK positive rates, levels of Ki67 expression, levels of MHC-I or CD8a in **d** (means ± SD). Statistical analysis was performed by two-tailed *t*-test. *P < 0.05; **P < 0.01; ***P < 0.001; ****P < 0.0001. **f** Kaplan–Meier curves of KPC mice were shown. The mice were treated with vehicle (Ctrl, *n* = 10 mice); Trametinib (Tra, *n* = 11 mice); Tat-Peptide-1 (Pep.1, *n* = 10 mice); the combination of Trametinib and Tat-Peptide-1 (Tra + Pep.1, *n* = 12 mice); chloroquine (CQ, *n* = 11 mice); the combination of Trametinib and chloroquine (Tra + CQ, *n* = 13 mice). **g** A model for oncogenic RINCAA.

To further validate the therapeutic potential of inhibiting PI4KB phosphorylation on S256 and T263, we lastly utilized a KPC mice model of pancreatic cancer, known for faithfully recapitulating human pancreatic cancer biology.^88–90^ Consistent with our findings in cancer cell lines and xenograft experiments, treatment with PI4KB-Peptide-1 blocked PI4KB phosphorylation on S256 and T263, and RINCAA in cancer tissues and, in combination with trametinib, effectively suppressed tumor growth and prolonged survival (Fig.10a–f). Notably, in contrast to chloroquine, PI4KB-Peptide-1 treatment alone decreased tumor growth and increased life span. In addition, the combination of PI4KB-Peptide-1 and trametinib exhibited superior efficacy than chloroquine plus trametinib in inhibiting tumor growth, preserving normal pancreatic tissue, and extending survival periods (Fig. 10d–f).

The tumor inhibition effect of PI4KB-Peptide-1 surpasses that of chloroquine in immune-competent mice but is similar in immune-deficient mice, suggesting that PI4KB-Peptide-1 positively regulates the immune response more effectively than chloroquine. Recent research demonstrated that autophagy in pancreatic cancer (with ~98% RAS mutation) impedes immune recognition of cancer cells by degrading MHC-I.^21^ To explore the possible involvement of immune regulation in the observed superior effect of PI4KB-Peptide-1, we analyzed MHC-I levels in the tumors (Fig. 10d–f). Both chloroquine and PI4KB-Peptide-1 increased MHC-I, consistent with previous findings of autophagy’s role in degrading MHC-I.^21^ However, only PI4KB-Peptide-1 enhanced CD8^+^ T cell infiltration (Fig. 10d–f). The lack of enhanced CD8^+^ T cell infiltration by chloroquine is likely due to chloroquine’s general inhibition of autophagy in T cells, as autophagy is essential for T cell function.^91–94^ In addition, chloroquine inhibits T cell function.^95,96^

Therefore, the tumor-specific inhibition of autophagy by PI4KB Peptide-1 provides a superior tumor-killing effect. This occurs by blocking the metabolic remodeling of cancer cells, enhancing MHC-I-mediated tumor antigen presentation, and minimally inhibiting CD8^+^ T cell activation. These data further support targeting PI4KB phosphorylation on S256 and T263 as a promising therapeutic strategy against RAS-mutation-driven cancers.

## Discussion

The present study identified a P38-ULK1-PI4KB axis as a special signaling cascade regulating a non-canonical form of autophagy in KRAS-mutated cells, which diverges from the nutrient sensing-modulated ULK1-PI3K signaling in starvation-regulated autophagy. Therefore, this study provides implications for how autophagy is specifically regulated under various disease states. Our data suggest a model wherein mutated KRAS activates P38, which in turn triggers the activation of ULK1. ULK1 then phosphorylates PI4KB on S256 and T263 and activates PI4KB to generate PI4P. Finally, WIPI2 acts as a PI4P effector and further recruits the lipidation machinery containing the ATG16L1/ATG12–ATG5 complex to promote the formation of non-canonical autophagic membranes. Differential requirement of PI-kinases, lipid transfer proteins and scramblases etc. results in the formation of atypical autophagosomes, termed RIMMBA, instead of the conventional double-membrane autophagosomes (Fig. 10). In addition, RIMMBA is different from conventional multivesicular body (MVB) (e.g., in the case of growth factor receptor downregulation) which usually mediated the action of the conventional ESCRT machinery (ESCRT-0 to ESCRT-III),^97^ in which atg8ylation has not been reported.

Although the PI3K complex is an essential player in starvation-induced autophagy by generating PI3P, which links upstream signaling to the membrane remodeling events,^28,29,98,99^ our study determined that the PI3K complex is not required for RINCAA. Non-canonical PI3K-independent autophagy has been reported elsewhere. Autophagosomes are detected in T lymphocytes and sensory neurons from *VPS34*^–/–^ mice and in glucose-starved cells treated with the VPS34 inhibitor wortmannin.^100,101^ PI3K is also not involved in a recently discovered unconventional secretion process regulated by multiple autophagic systems, as well as LC3-dependent EV loading and secretion.^102^ Nonetheless, in several cases, PI-phosphates other than PI3P are generated by specific PI-kinases to regulate the early steps of autophagosome biogenesis. For example, PIKfyve and its product PI5P regulate glucose starvation-induced autophagy^77^ and, in our case, PI4KB catalyzed the formation of PI4P for autophagosome biogenesis in RINCAA. Thus, WIPI2 is a shared effector for PI-phosphates by directly binding and activating the LC3 lipidation process. Therefore, it is likely that the generation of different types of PI-phosphates may represent regulatory diversification employed by different upstream signals converging on WIPI2 to induce autophagosome biogenesis.

PI4P, generated by different PI4Ks, plays multiple roles in different types of autophagy. PI4K2A catalyzes the formation of PI4P on the autophagosome, which facilitates ATG14 recruitment, and autophagosome–lysosome fusion, which is a late step in autophagy.^103–106^ PI4P on the autophagosome is targeted by SteA, which is a *Salmonella* pathogenic factor that blocks the formation of the autolysosome during xenophagy.^107^ PI4P generated by PI4K2A during secretory autophagy regulates the formation of non-canonical autophagosomes, which fuse with the RAB22A-positive early endosome to form a Rafeesome (RAB22A-mediated non-canonical autophagosome fused with an early endosome) that secretes STING via extracellular vesicles.^108^ PI4KA-regulated PI4P production controls autophagosome biogenesis in *Arabidopsis*.^109^ In addition to PI4K2A, PI4KB generates PI4P, which acts during the early stages of starvation-induced autophagy and RINCAA. During starvation, PI4KB is associated with ATG9A and ATG13 during the initial step of autophagy.^110^ Different from the published work, in our study, PI4KB replaces the PI3K complex to generate PI4P as a substitute for PI3P in RINCAA, which recruits WIPI2 (Figs. 2, 3).

Our study revealed that ATG2 and ATG9A are dispensable for RINCAA (Fig. 1m). The mechanisms underlying RIMMBA formation in the absence of these proteins remain to be determined. ATG2 functions as a lipid channel facilitating phospholipid delivery to the expanding autophagosome.^45,49,50^ Intriguingly, our findings indicate that the lipid channeling protein VPS13B is necessary for RINCAA (Fig. 1m). The involvement of Vps13 in autophagy has been reported in yeast.^52^ Moreover, one member of the VPS13 paralogues VPS13D was shown to regulate mitophagy in mammals.^53^ Therefore, VPS13B may act as a substitute for ATG2 in phagophore expansion. Additionally, ATG9A, a scramblase critical for autophagosome biogenesis, may be functionally replaced by other scramblases such as TMEM41B or VMP1 during RINCAA.^54–59^ Notably, VPS13s, TMEM41B, and VMP1 have been implicated in the regulation of autophagosome biogenesis in previous studies.^52–57^

The coordination between RINCAA and canonical autophagy within the cell remains incompletely understood. Our findings show that treatment with rapamycin and KRAS induction leads to an additive increase in LC3 lipidation, suggesting that these two autophagy pathways can operate concurrently within the cell (Supplementary information, Fig. S6f, g). However, it is plausible that competition may arise between these pathways when shared autophagic components become limited. Notably, under conditions of KRAS induction and starvation, ULK1 demonstrates selective activation of PI4KB and components of the PI3K complex (including Beclin-1), indicating that specific regulatory signals modulate ULK1’s role in coordinating these distinct forms of autophagy (Supplementary information, Fig. S6h).

RAS mutations occur at the highest rate ( ~25%) among cancers and are associated with several aggressive cancers.^111^ KRAS mutations are found in approximately 85% of all RAS-driven cancers and are thus regarded as the most frequently mutated oncogene in human cancer.^112–114^ Hyperactive RAS mutants are challenging to target due to the absence of drug-binding pockets on RAS proteins.^14^ Until recently, the only clinically effective inhibitors were AMG510 and MRTX849, which specifically target KRAS(G12C).^15,16^ Although several KRAS(G12X) inhibitors have been developed, comprehensive clinical evaluations are lacking, and patients can develop resistance through feedback mechanisms and genetic alterations in the RAS pathway.^115–117^ Therefore, identifying additional targets in RAS-mutated cancers is essential to improve therapeutic efficacy and address resistance. As an alternative approach, targeting the signaling pathway downstream of RAS has been extensively explored. Autophagy has been reported as a downstream effect of RAS signaling.^19,22^ High dependence on autophagy has been reported and combined inhibition of autophagy and ERK (activation of another downstream effect of RAS signaling) has been shown by multiple studies as a potential strategy against cancer with a RAS mutation.^18,39,40^ Nonetheless, physiological autophagy also plays essential roles in maintaining cellular homeostasis and protecting cells against stress, such as starvation.^3,118^ Therefore, it is necessary to identify the regulatory differences between physiological and RAS-related autophagy for specific and efficacious targeting of autophagy in cancers with RAS mutations. Our study identified differential regulation in the PI-phosphate generation step between RINCAA and starvation-induced autophagy. Mechanistic analysis identified a ULK1-regulated phosphorylation of K251–K269 region located on PI4KB as a special event of RINCAA (Fig. 5), which is in line with several studies showing ULK1 as a major regulator of autophagy in response to different cancer signals in tumor and the potency of ULK1 inhibitors against tumor.^40,119,120^

Importantly, phosphorylation of the PI4K-Peptide-1 occurred in higher rates in RAS-mutated CRC and several cancer cell lines with the RAS mutation (Fig. 7a–c), suggesting that this phosphorylation event may be a special signature of cancer with a RAS mutation. Hence, PI4KB S256 and T263 phosphorylation may be a specific target of non-canonical autophagy for treating cancer with a RAS mutation. In support of this idea, blocking PI4KB S256 and T263 phosphorylation with a competing substrate peptide or overexpressing a phosphorylation-deficient PI4KB-SA (which likely competes with endogenous phosphorylatable PI4KB) inhibits RINCAA and the growth of tumors with a KRAS mutation with equal or better effects compared to chloroquine (Figs. 6, 8, 9). While PI4KB plays a pivotal role in PI4P generation across various cellular processes, our findings indicate that S256 and T263 phosphorylation is not essential for PI4KB activity under normal conditions (Fig. 5i). This suggests a potential for minimal side effects when targeting PI4KB S256 and T263 phosphorylation.

In addition to promoting autophagy, RAS mutations have been reported to increase macropinocytosis, facilitating enhanced nutrient uptake in cancer cells.^121–123^ Although chloroquine has been shown to inhibit both autophagy and macropinocytosis, PI4KB depletion similarly inhibits both processes (Fig. 2; Supplementary information, Figs. S3, S6i, j). However, the more specific RINCAA inhibitor, PI4KB-Peptide-1, selectively blocks autophagy without affecting macropinocytosis (Supplementary information, Fig. S6k–n). Notably, despite this selectivity, PI4KB-Peptide-1 demonstrated superior antitumor efficacy compared to chloroquine, suggesting that preserving immune function and disrupting tumor metabolic remodeling are more critical for effectively treating RAS-mutant cancers than simply reducing nutrient scavenging. The high specificity of short peptides like PI4KB-Peptide-1 in targeting RINCAA within RAS-mutant cancers, while maintaining immune activity, underscores their potential as a promising anticancer approach. Future research should focus on developing small-molecule inhibitors targeting PI4KB phosphorylation at S256 and T263 and assessing their clinical effectiveness for enhanced therapeutic outcomes.

## Materials and methods

### Cells

Cells were maintained in Dulbecco’s modified Eagle’s medium (DMEM) (HEK293T, HCT116, SW620, A549, H1299, PANC1, MiaPaCa2, SW1990, MEF), or RPMI-1640 (FHC) with 10% FBS at 37 °C in 5% CO_2_. HEK293T cells stably expressing Flag-KRAS(G12V) were obtained by lentiviral infection followed by selection with antibiotics. For lentiviral transduction, HEK293T cells were transfected with plasmids (pMD2.G and psPAX2). Viruses were harvested at 60–72 h post-transfection. The viral supernatant was centrifuged at 600× *g* for 5 min to remove cell debris. The indicated cells were infected with the viral supernatant diluted in fresh medium (30% viral supernatant) with 10 µg/mL polybrene, which was exchanged to growth medium after 24 h. Transduced cells were selected and cultured in DMEM supplemented with 10% FBS, 150 μg/mL Zeocin (Sigma), and 15 μg/mL Blasticidin (Sigma). Target gene expression was confirmed via SDS-PAGE and immunoblot.

Primary antibodies used in this study are listed in Supplementary information, Table S2.

### Mice

The mouse experiments were approved by the Institutional Animal Care and Use Committees at Tsinghua University.

For xenograft studies, nude mice were purchased from Charles River (Beijing) and housed in ventilated cages in a temperature- and light-regulated room in a specific pathogen free (SPF) facility and received food and water ad libitum. Xenografted tumors were established by sub-cutaneous injection of 2 × 10^6^ HCT116 or SW620 cells resuspended in 100 μL of Matrigel (Yeasen, 40183ES10) into the flanks of upper thigh of 4-week-old male NOD/SCID mice. Treatment was then initiated with vehicle control (corn oil), trametinib at 1 mg/kg, Tat-Peptide-1 (Tat-Pep.1) at 40 mg/kg, chloroquine at 50 mg/kg, the combination of trametinib plus Tat-Pep.1 at the aforementioned dosages, or the combination of trametinib plus chloroquine at the aforementioned dosages via intraperitoneal injection twice a week. Tumors were measured twice weekly via calipers and tumor volume was calculated by volume = 4/3 × π × (((length + width)/2)/2).^3^ More than 12 tumors per group were analyzed. The tumors were removed, photographed, and weighed, and the average weights of the tumors were calculated. Significance of difference in tumor size was calculated by a two-tailed *t*-test.

KPC mice were a gift from Charles J. David (Tsinghua University). Age- and sex-matched (except where indicated otherwise) male and female mice of the genotype were generated as littermates for use in experiments. Treatment was then initiated with vehicle control (corn oil), trametinib at 1 mg/kg, Tat-Peptide-1 (Tat-Pep.1) at 40 mg/kg, the combination of trametinib plus Tat-Pep.1 at the aforementioned dosages, chloroquine at 40 mg/kg or the combination of trametinib plus chloroquine at the aforementioned dosages via intraperitoneal injection three times a week.

### Plasmids, siRNA oligos, and transfection

Human KRAS(G12V), KRAS(G12D), or KRAS(G12C) was cloned into pLenti-CMV vector. The mCherry-pHluorin-LC3B, mCherry-ATG4B-C74S, OSBP-PH, WIPI2-GST

Flag-ULK1, Myc-ULK1, HA-ULK1, EGFP-PI4KB, HA-PI4KB and GFP-Pep.1 plasmids were generated by PCR and ligation. HA-PI4KB-SA, HA-PI4KB-D656 and Myc-ULK1-K46I plasmids were generated by site mutagenesis PCR. The siRNAs and shRNAs are listed in Supplementary information, Table S3. Transfection of PI4P (Avanti Polar Lipids) was performed using Unlabeled Shuttle PIP Carrier 3 (Echelon Biosciences). Transfection of plasmids was performed using PEI (Polysciences, Inc.) for HEK293T and X-tremeGENE HP (Roche) for FHC according to the manufacturers’ protocols. The siRNA transfection was performed with Lipofectamine RNAiMAX (Invitrogen) according to the manufacturer’s protocol.

### Reagents

Doxycycline Hyclate was purchased from Beyotime (Cat# ST039B). Inhibitors including Bafilomycin, wortmannin, AMG510, SAR405, PIK-III, VPS34-IN1, PIK93, SB203580, JNK-IN-8, FR180204, MK2206, RBC8, SBI-0206965 were purchased from Selleck (Cat# S1413, S2578, S8830, S7682, S7683, S7980, S1489, S1076, S4901, S7524, S1078, S7606, and S7885, respectively). T-00127-HEV1 was purchased from MedChemExpress (Cat# HY-108313). YM201636 was purchased from Topscience, USA (Cat# T6110). PI4P was purchased from Echelon Biosciences (Cat# p4016-2). Digitonin was purchased from BIOSYNTH (Cat# D-3200). CIP was purchased from Sigma (Cat# P4978). Cycloheximide was purchased from Cell Signaling Technology (Cat# 2112S).

### Peptide synthesis

l-amino acid peptides were synthesized by Scilight Biotechnology LLC. The Tat-peptide-1 (Tat-Pep.1) peptide sequence, YGRKKRRQRRRGGELPSLSPAPDTGLSPSK, consisted of 11 amino acids from the Tat PTD at the N-terminus, a GG linker to increase flexibility, and at the C-terminus, 17 amino acids derived from PI4KB (253–269). The control peptide (Tat-scrambled) consisted of the Tat protein transduction domain, a GG linker, and a scramble sequence (YGRKKRRQRRRGGVGNDFFINHETTGFATEW).

### Immunofluorescence

The cells were incubated with 4% cold paraformaldehyde for 20 min at room temperature. The cells were further permeabilized with 0.1% Triton X-100 diluted in phosphate-buffered saline (PBS) at room temperature for 10 min, followed by blocking with 10% FBS diluted in PBS for 1 h and the primary antibody incubation for 1 h at room temperature. Cells were washed three times with PBS, followed by the secondary antibody incubation for 1 h at room temperature.

For the tissue immunofluorescence, 5 μm sections of the paraffin-embedded tumors were kept at 60 °C for 24 h in the oven followed by deparaffinization with xylene and hydration with an ethanol gradient (100%–70%). After successively incubating with antigen retrieval solution (ZSGB Biotechnology Company; Beijing, China) and 3% H_2_O_2_ for 30 min, the slides were rinsed with water and incubated with the primary antibody overnight at 4 °C. The next day, the slides were rinsed and incubated with the corresponding secondary antibody for 1 h at room temperature.

Fluorescence images were acquired using the Olympus FV3000 confocal microscope. Quantification was performed using ImageJ.

### HaloTag-LC3B processing assay

HEK293T cells stably expressing the Tet-on KRAS(G12V) system and HaloTag-LC3B were used for autophagic flux analysis. After 36 h of induction with Dox (final concentration, 1 µg/mL), the cells were pulse-labeled for 20 min with 100 nM tetramethyl rhodamine (TMR)-conjugated ligand (Promega, G8251) in nutrient-rich medium. Following two washes with PBS, the cells were further incubated for 2 h in fresh medium containing Dox (final concentration 1 µg/mL) alone or in combination with 100 nM Baf A1. After incubation, cells were lysed and 20 µg of protein per sample was subjected to SDS-PAGE. For in-gel fluorescence imaging, the gel was immediately imaged using ChemiDoc Imaging System (Bio-Rad) after SDS-PAGE. For immunoblotting, proteins were transferred from the SDS-PAGE gel to Immobilon-P polyvinylidene difluoride (PVDF) membranes (Millipore, IPVH00010). Following incubation with the appropriate antibodies, the signals were detected using chemiluminescent HRP substrate and ChemiDoc Imaging System (Bio-Rad). Band intensities were quantified using the Gel Analyzer tool in the open-source image processing software Fiji.

### CHX chase assay

HEK293T cells stably expressing the Tet-on KRAS(G12V) system were treated with 50 mg/mL CHX, with or without 0.5 mg/mL Baf A1 as indicated and were collected at each indicated time point for immunoblot analysis.

### Co-IP and immunoblot

For co-IP, the cells were lysed on ice for 30 min in the IP buffer (50 mM Tris/HCl, pH 7.4, 150 mM NaCl, 1 mM EDTA, 0.5% NP40, 10% glycerol) with protease inhibitor mixture, and the lysates were cleared by centrifugation. The resulting supernatants were incubated with indicated antibody-conjugated agarose beads and rotated for 3 h at 4 °C. Then the agarose beads were washed five times with the IP buffer and checked by immunoblot.

### Immunoprecipitation and in vitro kinase assay

Cells expressing indicated proteins were lysed in ice-cold lysis buffer (20 mM Tris, pH 7.5, 150 mM NaCl, 0.3% (vol/vol) Triton X-100, 5 mM EDTA) supplemented with Complete EDTA-free protease inhibitor cocktail (Roche) and PhosStop-phosphatase inhibitor cocktail (Roche). The lysates were centrifuged, and the supernatant was incubated with indicated antibody-conjugated agarose beads at 4 °C for 1 h. Following incubation, the beads were washed three times with the lysis buffer (above-mentioned) and then once with kinase reaction buffer (20 mM HEPES, pH 7.5, 20 mM MgCl_2_, 25 mM beta-glycerophosphate, 2 mM dithiothreitol, 100 µM sodium orthovanadate). Subsequently, the beads were incubated in a final volume of 20 µL reaction buffer containing 20 µM ATP and 5 µg purified GFP-PI4KB protein at 30 °C for 15 min. The reactions were stopped by adding 5× SDS sample buffer and heating to 65 °C for 5 min. Proteins were then transferred to PVDF membranes for analysis by immunoblotting.

### Dot blot

For determination of PI4P, total lipids were extracted from HEK293T cells. Each 300 mL cell suspension was incubated with 1.2 mL chloroform/methanol solution (chloroform: methanol = 1:2) and vortexed for 30 s and shaken for 1 h at 180 rpm at 37 °C. The chloroform phase was collected and evaporated by a stream of nitrogen gas over the lipid solution and further dried in an incubator at 37 °C for 1 h. Dried lipids were suspended in absolute ethyl alcohol. The lipids solution was measured and used as a standard (PC) to normalize lipids concentration. The lipids were applied to the nitrocellulose membrane (Millipore; Bedford, MA, USA) and left to dry for 1 h. The membrane was incubated with the PI4P antibody overnight at room temperature. On the next day, the membrane was washed and incubated with an anti-mouse antibody (diluted 1:1000 in the blocking solution) for 1 h. Finally, the PI4P level recognized by the antibody was revealed by treatment with chemiluminescent.

For determination of WIPI2 binding to lipids, purified WIPI2 was used to overlay membrane-immobilized phospholipid membranes (PIP Strips, Echelon, Cat# P-6001). Anti-WIPI2 and anti-mouse secondary antibodies were used to detect the proteins by scanning with the ChemiDoc Imaging Systems.

### qRT-PCR

Total RNA was isolated from different cell lines using TRIzol reagent (Beyotime, Cat# R0016) according to the manufacturer’s instructions. Equal amounts of RNA were reverse transcribed into cDNA using a Revert Aid First Strand cDNA synthesis kit (Abclonal, E047-01B) according to the manufacturer’s instructions. Quantitative PCR was performed using an ABI Step One Plus system. The PCR reactions were carried out in 10 μL reactions using SYBR Green PCR master mix (Abclonal, RK20429) and 0.5 μM specific primers. The primers used for PCR are shown in Supplementary information, Table S3.

### EM and APEX2-DAB staining

For 3,3′-diaminobenzidine (DAB) staining and preparation of cultured cells for EM, 293T-KRAS(G12V) cells were transfected with APEX2-LC3B and concurrently induced with Dox (final concentration, 1 µg/mL). After 36 h of induction, the cells were fixed using 2% glutaraldehyde in buffer (100 mM sodium cacodylate with 2 mM CaCl_2_, pH 7.4), then quickly moved to ice. Cells were kept between 0 °C and 4 °C for all subsequent steps until resin infiltration. After 30–60 min, cells were rinsed 5 times for 2 min each with chilled buffer, treated for 5 min in buffer containing 20 mM glycine to quench unreacted glutaraldehyde followed by 2 min rinse for 5 times with chilled buffer. A freshly diluted solution of 0.5 mg/mL (1.4 mM) DAB tetrahydrochloride or the DAB free base (Sigma) dissolved in HCl was combined with 0.03% (v/v) (10 mM) H_2_O_2_ in chilled buffer, and the solution was added to cells for 5 min. The generation of reaction product could be monitored by transmitted LM. To halt the reaction, the DAB solution was removed, and cells were rinsed 5× 5 min with chilled buffer. Post-fixation staining was performed with 2% osmium tetroxide for 30 min in chilled buffer. Cells were rinsed 5 times for 2 min each with chilled distilled water and placed in chilled 2% aqueous uranyl acetate overnight. The samples were then dehydrated in a cold-graded ethanol series (20%, 50%, 70%, 90%, 100%, 100%) 2 min each, rinsed once in room temperature anhydrous ethanol to avoid condensation, and infiltrated in Durcupan ACM resin (Electron Microscopy Sciences) using 1:1 (v/v) anhydrous ethanol and resin for 30 min, and 100% resin twice for 1 h each. Finally, the samples were embedded into fresh resin and polymerized in a vacuum oven at 60 °C for 48 h. DAB-stained areas of embedded cultured cells were identified by transmitted light, and the areas of interest were sawed out using a jeweler’s saw and mounted on dummy acrylic blocks with cyanoacrylic adhesive (Krazy Glue, Elmer’s Products). The coverslip was carefully removed, the block was trimmed, and ultrathin (80 nm thick) sections were cut using an ultramicrotome (Leica Ultracut UTC6). Samples were imaged under the HT-7800 120 kv transmission electron microscope.

### In situ CLEM/electron tomography ET

For sample preparation, FHC cells expressing BFP-KRAS-G12V, EGFP-tagged OSBP-PH and mCherry-LC3B were seeded onto ultraviolet-sterilized ELI grids (T11012ss-ELI, TIANLD) and cultured in complete DMEM supplemented with 10% FBS and 1% penicillin and streptomycin. After 48 h of culture with 5% CO_2_ at 37 °C, the grids were subjected to plunge freezing by backside blotting and vitrification using a Leica EM GP (Leica Microsystems). Next, the cryo-vitrified grid was assembled into a FEI AutoGrid (Thermo Fisher Scientific, USA) for the subsequent cryo-transfer and imaging.

For the HOPE-SIM screening, cryo-SIM images were acquired on the HOPE-SIM imaging system with a 100× 0.9 dry objective (Nikon), a laser combiner of three lasers with wavelengths of 405 nm (50 mW, OBIS 405LX, Coherent), 488 nm (200 mW, SAPPHIRE 488-200 CW, Coherent), and 561 nm (200 mW, SAPPHIRE 561-200 CW, Coherent) and a high-sensitivity sCMOS camera (Prime 95B, Photometrics). Each raw image has a pixel size of 120 nm and a field of view of 1200 × 1200 pixels. Then, the final raw image stack has a voxel size of 120 nm × 120 nm × 250 nm.

For cryo-FIB milling, cryo-lamellae were prepared by cryo-FIB milling using the FEI Helios NanoLab 600i (Thermo Fisher Scientific, USA). Cryo-FIB milling was accurately navigated by monitoring the real-time fluorescence signal of the target molecule by using ELI trifocal microscope. CLEM correlation operations were performed in 3D-View software.

For cryo-ET data collection, the cryo-lamellae were loaded into a FEI Titan Krios G3i (Thermo Fisher Scientific, USA) that was equipped with a Gatan GIF K2 4k × 4k camera (Gatan, Inc., Warrendale, PA, USA) and operated at 300 kV in low-dose mode. Tilt series were collected bidirectionally with SerialEM software using a tilt range of −55° to 50° in 2° intervals, nominal magnification of 33,000×, defocus of −6 μm. All procedures for cryo-EM imaging were performed under low-dose conditions.

For data processing, image movies were processed by motion correction and CTF estimation in Warp. The produced tilt series were aligned using IMOD.^124^ Amira 10.0 (Thermo Fisher Scientific, USA) was applied to segment structures of the cryo-ET images. The characteristics of the structures were labeled manually and colored differently. All movies were generated in Imaris 10.0 (Oxford Instruments).

### MS and phos-tag gel analysis

HEK293T cells expressing HA-PI4KB with or without Myc-ULK1 were cultured on three 10-cm dishes to 100% confluency. The small-molecule kinase inhibitor SBI-0206965 was added to the culture to inhibit the ULK1. Then agarose coupled with anti-HA antibodies was employed to immunoisolate the HA-PI4KB and eluted with the HA peptide. Purified HA-PI4KB was analyzed by SDS–PAGE and visualized by Coomassie Brilliant Blue staining. Specific HA-PI4KB bands were subjected to MS analysis to identify post-translational modifications including phosphorylation. MS was performed by the Protein Chemistry and Proteomics Center at Tsinghua University. Phos-tag gel analysis (Wako, 199-17391) was applied to confirm the phosphorylation of PI4KB according to the manufacturer’s instructions.

### Liposome floatation and pelleting assay

1-palmitoyl-2-oleoyl-glycero-3-phosphocholine (POPC), 1-palmitoyl-2-oleoyl-sn-glycero-3-phospho-L-serine (POPS), 1,2-dioleoyl-sn-glycero-3-phosphoethanolamine (DOPE), cholesterol, PI3P, PI4P, and PI5P were purchased from Avanti Polar Lipids. Lipids were mixed with a ratio of 4:2.5:2.5:10 for POPC:POPS:DOPE:cholesterol plus 1% PI3P, PI4P, or PI5P. The preparation of small unilamellar vesicles (SUVs) was described previously,^125^ with the lipid mixture above. The lipid mixtures were dried with a nitrogen stream and further dried for 1 h at 37 °C. The lipid film was then hydrated sufficiently with the HEPES buffer (20 mM HEPES, pH 7.4; 150 mM NaCl) and subjected to 10 cycles of freezing in the liquid nitrogen and thawing in a 42 °C water bath. Finally, the liposomes were extruded 20 times through the polycarbonate film with a specific pore size to generate the SUVs.

For the floatation assay, purified WIPI2 was added into 120 μL SUVs solution with the described concentration and incubated for 30 min at room temperature. In order to remove the free proteins, a membrane floatation procedure was performed as described before.^125^ Briefly, 480 μL 50% OptiPrep was added to the 120 μL solution above. The mixture was overlaid successively with 480 μL 30% OptiPrep and 90 μL HEPES buffer, then centrifuged at 100,000× *g* for 2 h. The 60 μL top fraction was collected. 1 μL of the top fraction was taken to measure the PC concentration with a microplate spectrophotometer. The rest was added with 5× SDS loading buffer and analyzed by immunoblot.

### Cell proliferation assay

Cell proliferation was assessed using a CCK-8 assay kit (Dojindo Molecular Technologies). Briefly, cells were seeded in 96-well plates. 10 μL CCK-8 solution was added to each well containing 100 μL culture medium and incubated for 2 h at 37 °C. The absorbance was measured at 450 nm wavelength using an ELISA plate reader. For the cell proliferation assays, cell growth was analyzed on the first and the fourth day.

### IHC staining

Pancreata or tumors were dissected and fixed in 4% paraformaldehyde in PBS and embedded in paraffin. 5 μm sections were prepared and stained with hematoxylin and eosin (H&E). For IHC, 5 μm sections of the paraffin-embedded tumors or pancreata were kept at 60 °C for 24 h in the oven followed by deparaffinization with xylene and hydration with an ethanol gradient (100%−70%). After successively incubating with antigen retrieval solution (ZSGB Biotechnology Company; Beijing, China) and 3% H_2_O_2_ for 30 min, the slides were rinsed with water and incubated with the primary antibody overnight at 4 °C. On the next day, the slides were rinsed and incubated with the corresponding secondary antibody (ZSGB Biotechnology Company; Beijing, China) for 30 min followed by DAB and H&E staining, respectively.

Tissue microarrays containing tumor tissues and their corresponding adjacent normal tissues from colon cancer and rectum cancer were obtained from Shanghai Zhuoli Biotechnology Co., Ltd. The samples were paraffin-embedded. Patient consent and approval from the Institutional Research Ethics Committee were obtained for the usage of these clinical materials for research purposes. Paraffin-embedded tissue sections were prepared according to standard methods, and the expression of p-PI4KB (1:1000 dilution) or PI4KB (1:500 dilution) was detected by IHC staining. The slides were assessed by pathologists who were blinded to the experimental results and patient outcomes.

### IHC scoring

A modified labeling score (H score) is calculated by using percentage of positively stained cancer cells and their intensity per tissue core. The intensity of staining was evaluated by an immunostaining score, which was calculated as the sum of the proportion and intensity of the stained tumor cells. Briefly, a proportion score, which represented the estimated proportion of positively stained tumor cells (0, none; 1, < 1/100; 2, > 1/100 to < 1/10; 3, > 1/10 to < 1/3; 4, > 1/3 to < 2/3, and 5, > 2/3.), was obtained. Next, an intensity score, which indicated the average intensity of positively stained tumor cells (0, none; 1, weak; 2, intermediate; and 3, strong), was obtained. The proportion and intensity scores were then added to obtain a total score, which ranged from 0 to 8.

### Statistical analysis

The ways of quantification of each experiment have been provided in the method details. The statistical information of each experiment, including the statistical methods, the *P* values and numbers (*n*), were shown in the figures and corresponding legends. Statistical analyses were performed with GraphPad Prism.

## Supplementary information

Fig. S1

Fig. S2

Fig. S3

Fig. S4

Fig. S5

Fig. S6

Supplementary information, Video S1

Table. S1

Table. S2

Table. S3
