# Supplementary material for: Oncogenic RAS induces a distinctive form of non-canonical autophagy mediated by the P38-ULK1-PI4KB axis
Source: Cell Res. 2025 Mar 7;35(6):399–422. doi: 10.1038/s41422-025-01085-9 (PMC12134136; doi:10.1038/s41422-025-01085-9)
Supplement: Supplementary file 1 — Fig. S1 [file 41422_2025_1085_MOESM1_ESM.pdf]

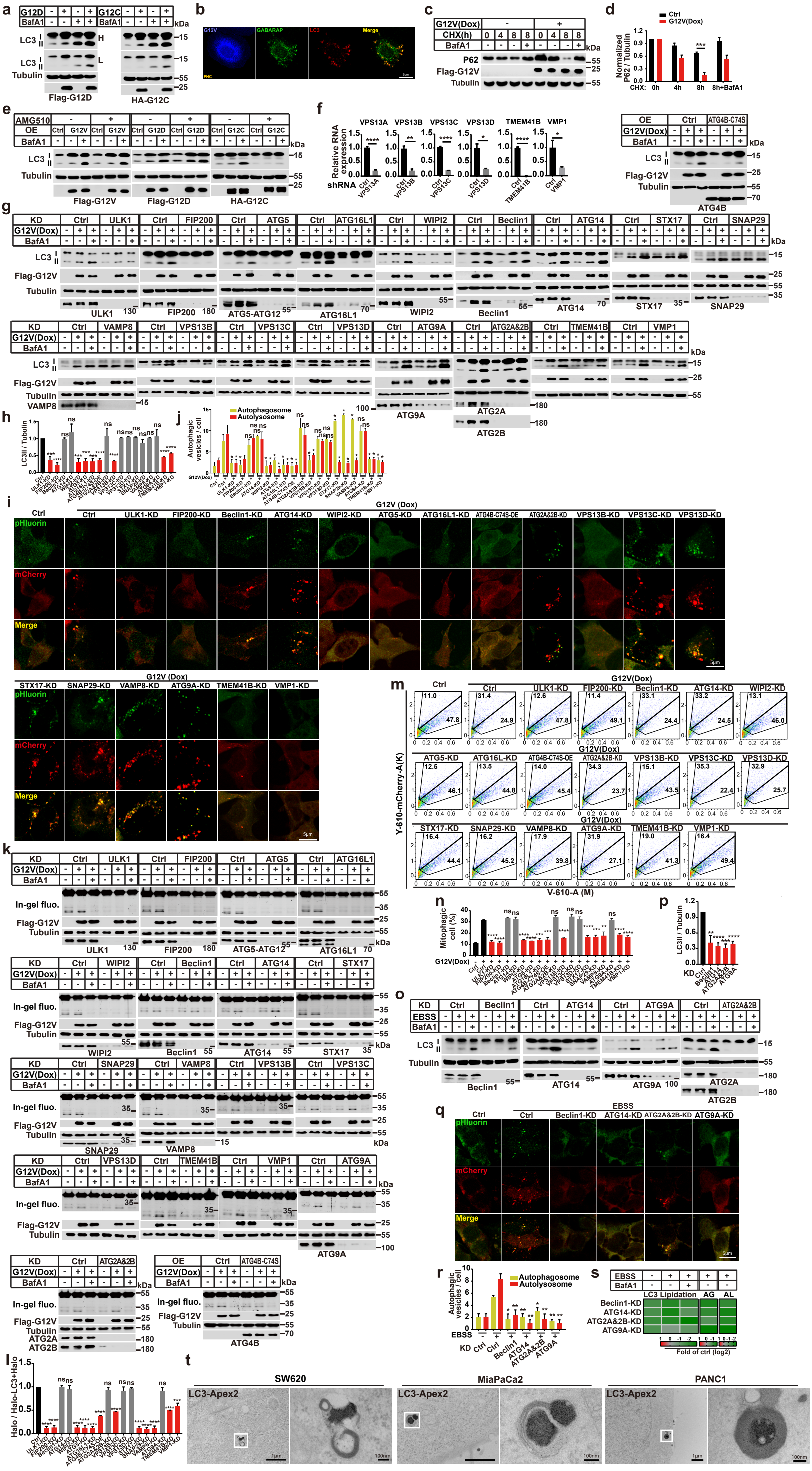
a
G12D
BafA1
G12C
LC3 I II
H
L
Tubulin
Flag-G12D
HA-G12C
kDa
b
G12V
GABARAP
LC3
Merge
c
G12V(Dox)
CHX(h)
BafA1
P62
Flag-G12V
Tubulin
d
Ctrl
G12V(Dox)
Normalized P62 / Tubulin
CHX: 0h 4h 8h 8h+BafA1
e
AMG510
OE
LC3 I II
Tubulin
Flag-G12V
Flag-G12D
HA-G12C
f
VPS13A VPS13B VPS13C VPS13D TMEM41B VMP1
Relative RNA expression
shRNA
OE Ctrl ATG4B-C74S
G12V(Dox)
BafA1
Flag-G12V
Tubulin
ATG4B
g
KD
Ctrl ULK1 FIP200 ATG5 ATG16L1 WIPI2 Beclin1 ATG14 STX17 SNAP29
Ctrl VAMP8 VPS13B VPS13C VPS13D ATG9A ATG2A&2B TMEM41B VMP1
ATG5-ATG12
ATG9A
ATG2A
ATG2B
h
LC3II / Tubulin
j
Autophagosome
Autolysosome
Autophagic vesicles / cell
i
Ctrl
G12V (Dox)
ULK1-KD FIP200-KD Beclin1-KD ATG14-KD WIPI2-KD ATG5-KD ATG16L1-KD ATG4B-C74S-OE ATG2A&2B-KD VPS13B-KD VPS13C-KD VPS13D-KD
pHluorin
mCherry
Merge
STX17-KD SNAP29-KD VAMP8-KD ATG9A-KD TMEM41B-KD VMP1-KD
5μm
m
Y-610-mCherry-A(K)
V-610-A (M)
k
In-gel fluo.
Flag-G12V
Tubulin
n
Mitophagic cell (%)
p
LC3II / Tubulin
o
EBSS
BafA1
Ctrl Beclin1 ATG14 ATG9A ATG2A&2B
q
EBSS
Ctrl Beclin1-KD ATG14-KD ATG2A&2B-KD ATG9A-KD
r
Autophagosome
Autolysosome
Autophagic vesicles / cell
s
EBSS
BafA1
LC3 Lipidation AG AL
Beclin1-KD
ATG14-KD
ATG2A&2B-KD
ATG9A-KD
Fold of ctrl (log2)
l
Halo / Halo-LC3+Halo
t
SW620
MiaPaCa2
PANC1
LC3-Apex2
1μm
100nm

Figure. S1 Characterization of KRAS(G12V)-induced autophagy

a. Immunoblot analysis of the LC3 lipidation of the cell lysates from the control cells and the cells transfected with KRAS(G12D) or KRAS(G12C) plasmids.

b. Immunofluorescence analysis the colocalization of GABARAP and LC3 in the KRAS(G12V)-expressing FHC cells.

c. Immunoblot analysis of turnover of P62 in CHX chase assay from KRAS(G12V) HEK293T cells treated with or without Dox.

d. Quantification of the ratio of normalized P62 to tubulin with the 0h point set as 1.00 analyzed in **c** (mean ± SEM). Three independent experiments were performed for the statistical analysis (two-tailed t-test). ***, $P < 0.001$.

e. Immunoblot analysis of the LC3 lipidation of the cell lysates from the control or KRAS(G12V), KRAS(G12D), KRAS(G12C) cells treated with 10 μM AMG510 for 1.5 h in the absence or presence of 500 nM Bafilomycin A1 for 1.5 h.

f. Relative RNA expression of cells transfected with control or shRNAs against VPS13B, VPS13C, VPS13D, TMEM41B and VMP1 (mean ± SEM). Three independent experiments were performed for the statistical analysis (two-tailed t-test). *, $P < 0.05$; **, $P < 0.01$; ***, $P < 0.001$; ****, $P < 0.0001$.

g. Immunoblot analysis of the LC3 lipidation from KRAS(G12V)-cells with control or knockdown of ULK1, FIP200, ATG5, ATG16L1, WIPI2, Beclin1, ATG14, STX17, SNAP29, VAMP8, VPS13B, VPS13C, VPS13D, ATG9A, ATG2A&B, TMEM41B, VMP1 or ATG4B-C74S overexpression in the absence or presence of 500 nM Bafilomycin A1 for 1.5 h.

h. Quantification of the ratio of lipidated LC3 to tubulin with the control set as 1.00 (control cells without Bafilomycin A1) analyzed in **g** (mean ± SEM). Three independent experiments were performed for the statistical analysis (two-tailed t-test). ***, $P < 0.001$; ****, $P < 0.0001$.

i. Immunofluorescence of the mCherry-pHluorin-LC3B in the KRAS(G12V)-expressing HEK293T cells with control or knockdown of ULK1, FIP200, Beclin1, ATG14, WIPI2, ATG5, ATG16L1, ATG2A&B, VPS13B, VPS13C, VPS13D, STX17, SNAP29, VAMP8, ATG9A, TMEM41B, VMP1 or overexpressing ATG4B-C74S. Representative cell images are shown. Scale bar sizes are indicated

in the image.

j. Quantification of the yellow (RFP^+GFP^+) and Red (RFP^+GFP^-) LC3 puncta. Data are represented as mean ± SEM. Three independent experiments (50 cells for each group/experiment) were performed for the statistical analysis (two-tailed t-test). *, $P < 0.05$.

k. Immunoblotting and in-gel fluorescence detection of in the KRAS(G12V) and control cells stably expressing HaloTag (Halo)-LC3B pulse-labeled for 20 min with 100 nM tetramethyl rhodamine (TMR)-conjugated ligand in nutrient-rich medium with control or knockdown of ULK1, FIP200, Beclin1, ATG14, WIPI2, ATG5, ATG16L1, ATG2A&B, VPS13B, VPS13C, VPS13D, STX17, SNAP29, VAMP8, ATG9A, TMEM41B, VMP1 or ATG4B-C74S overexpression in the absence or presence of 500 nM Bafilomycin A1 for 1.5 h.

l. Quantification of results shown in **k**. Halo-TMR band intensity was normalized by the sum of the band intensities Halo-TMR-LC3B and Halo-TMR, and the control set (KRAS(G12V) HEK293T cells with ligand without Bafilomycin A1) as 1.00. Three independent experiments were performed for the statistical analysis (two-tailed t-test). ***, $P < 0.001$; ****, $P < 0.0001$.

m. FACS analysis of the control and KRAS(G12V) cells co-expressing mt-Keima and Parkin with control or knockdown of ULK1, FIP200, Beclin1, ATG14, WIPI2, ATG5, ATG16L1, ATG2A&B, VPS13B, VPS13C, VPS13D, STX17, SNAP29, VAMP8, ATG9A, TMEM41B, VMP1 or ATG4B-C74S overexpression using V610 and Y610-mCherry detectors (Beckman CytoFLEX LX). The FACS results are representative of at least Three independent independent experiments.

n. Quantification of results shown in **m**. The percentage of cells with mitophagy based on Y610-mCherry/V610. Data are represented as mean ± SEM. Three independent experiments were performed for the statistical analysis (two-tailed t-test). **, $P < 0.01$; ***, $P < 0.001$; ****, $P < 0.0001$.

o. Immunoblot analysis of the LC3 lipidation from cells transfected with control or knockdown of Beclin1, ATG14, ATG9A, or ATG2A&B in starvation-induced autophagy by EBSS treatment in the absence or presence of 500 nM Bafilomycin A1 for 1.5 h.

p. Quantification of the ratio of lipidated LC3 to tubulin with the control set as 1.00 (KRAS(G12V) cells with Bafilomycin A1) analyzed in **o** (mean ± SEM). Three independent experiments were performed for the statistical analysis (two-tailed t-test). **, $P < 0.01$; ***, $P < 0.001$; ****, $P < 0.0001$.

q. Immunofluorescence of HEK293T cells expressing mCherry-pHluorin-LC3B with control or knockdown of Beclin1, ATG14, ATG9A and ATG2A&B in starvation-induced autophagy by EBSS treatment. Representative cell images are shown. Scale bar sizes are indicated in the image.

r. Quantification of the yellow ($RFP^{+}GFP^{+}$) and Red ($RFP^{+}GFP^{-}$) LC3 puncta. Data are represented as mean ± SEM. Three independent experiments (50 cells for each group/experiment) were performed for the statistical analysis (two-tailed t-test). *, $P < 0.05$, **, $P < 0.01$.

s. Heatmap showing the changes of LC3 lipidation (the ratio of lipidated LC3 to tubulin with the control set as 1.00, related to Supplementary information, Fig. S1m, n) and autophagic flux by the tandem fluorescent LC3 system (AG and AL, the control set as 1.00, related to Supplementary information, Fig. S1o, p) in the control and EBSS group with knockdown Beclin1, ATG14, ATG2A&2B, or ATG9A, respectively. Color represents the $\log_2$ (fold) of counts per samples.

t. Electron microscopy images of APEX2-labeled LC3 and the autophagosomes in multiple cancer cell lines with RAS mutations. Scale bar sizes are indicated in the image.
