# Supplementary material for: Oncogenic RAS induces a distinctive form of non-canonical autophagy mediated by the P38-ULK1-PI4KB axis
Source: Cell Res. 2025 Mar 7;35(6):399–422. doi: 10.1038/s41422-025-01085-9 (PMC12134136; doi:10.1038/s41422-025-01085-9)
Supplement: Supplementary file 2 — Fig. S2 [file 41422_2025_1085_MOESM2_ESM.pdf]

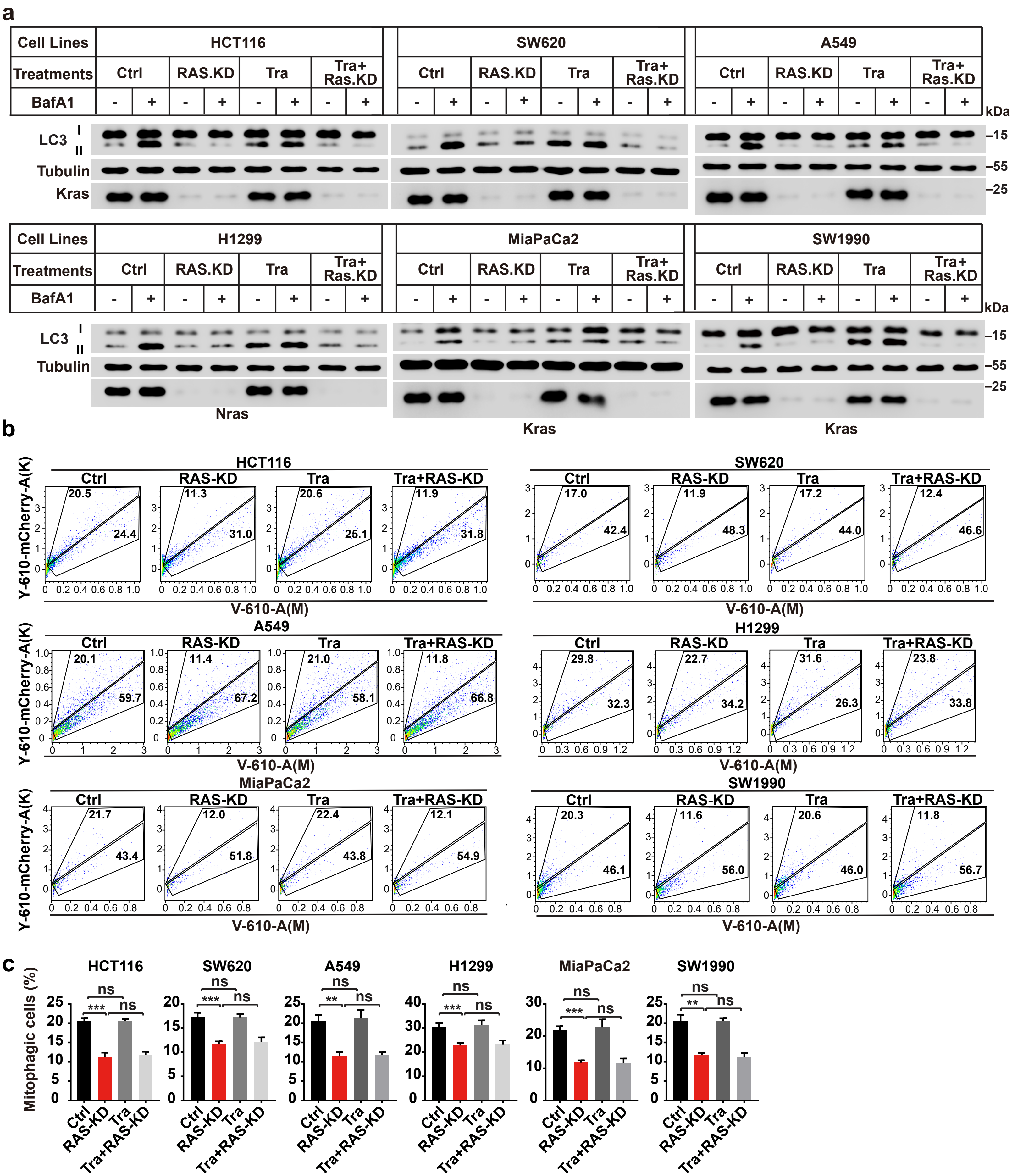
a
Cell Lines
HCT116
SW620
A549
Treatments
Ctrl
RAS.KD
Tra
Tra+ Ras.KD
BafA1
kDa
LC3
I
II
Tubulin
Kras
–15
–55
–25
H1299
MiaPaCa2
SW1990
Nras
Kras
b
Y-610-mCherry-A(K)
V-610-A(M)
Ctrl
RAS-KD
Tra
Tra+RAS-KD
HCT116
20.5
24.4
11.3
31.0
20.6
25.1
11.9
31.8
SW620
17.0
42.4
11.9
48.3
17.2
44.0
12.4
46.6
A549
20.1
59.7
11.4
67.2
21.0
58.1
11.8
66.8
H1299
29.8
32.3
22.7
34.2
31.6
26.3
23.8
33.8
MiaPaCa2
21.7
43.4
12.0
51.8
22.4
43.8
12.1
54.9
SW1990
20.3
46.1
11.6
56.0
20.6
46.0
11.8
56.7
c
Mitophagic cells (%)
HCT116
SW620
A549
H1299
MiaPaCa2
SW1990
ns
***
**
Ctrl
RAS-KD
Tra
Tra+RAS-KD

Figure. S2 RAS knockdown led to decreased autophagosome biogenesis and autophagic flux

a. Immunoblot analysis of LC3 lipidation of HCT116, SW620, A549, H1299, MiaPaCa-2 and SW1990 cells with or without RAS knockdown and Trametinib (Tra, 1 μM) treatment for 24 h in the absence or presence of 500 nM Bafilomycin A1 for 1.5 h.

b. FACS analysis of HCT116, SW620, A549, H1299, MiaPaCa-2 and SW1990 cells co-expressing mt-Keima and Parkin with or without RAS knockdown and Trametinib (Tra, 10 μM) treatment using V610 and Y610-mCherry detectors (Beckman CytoFLEX LX). The FACS results are representative of at least Three independent independent experiments.

c. The percentage of cells with mitophagy based on Y610-mCherry/V610 calculated for **b**. Data are represented as mean ± SEM. Three independent experiments were performed for the statistical analysis (two-tailed t-test). **, $P < 0.01$; ***, $P < 0.001$.
