# Supplementary material for: Oncogenic RAS induces a distinctive form of non-canonical autophagy mediated by the P38-ULK1-PI4KB axis
Source: Cell Res. 2025 Mar 7;35(6):399–422. doi: 10.1038/s41422-025-01085-9 (PMC12134136; doi:10.1038/s41422-025-01085-9)
Supplement: Supplementary file 3 — Fig. S3 [file 41422_2025_1085_MOESM3_ESM.pdf]

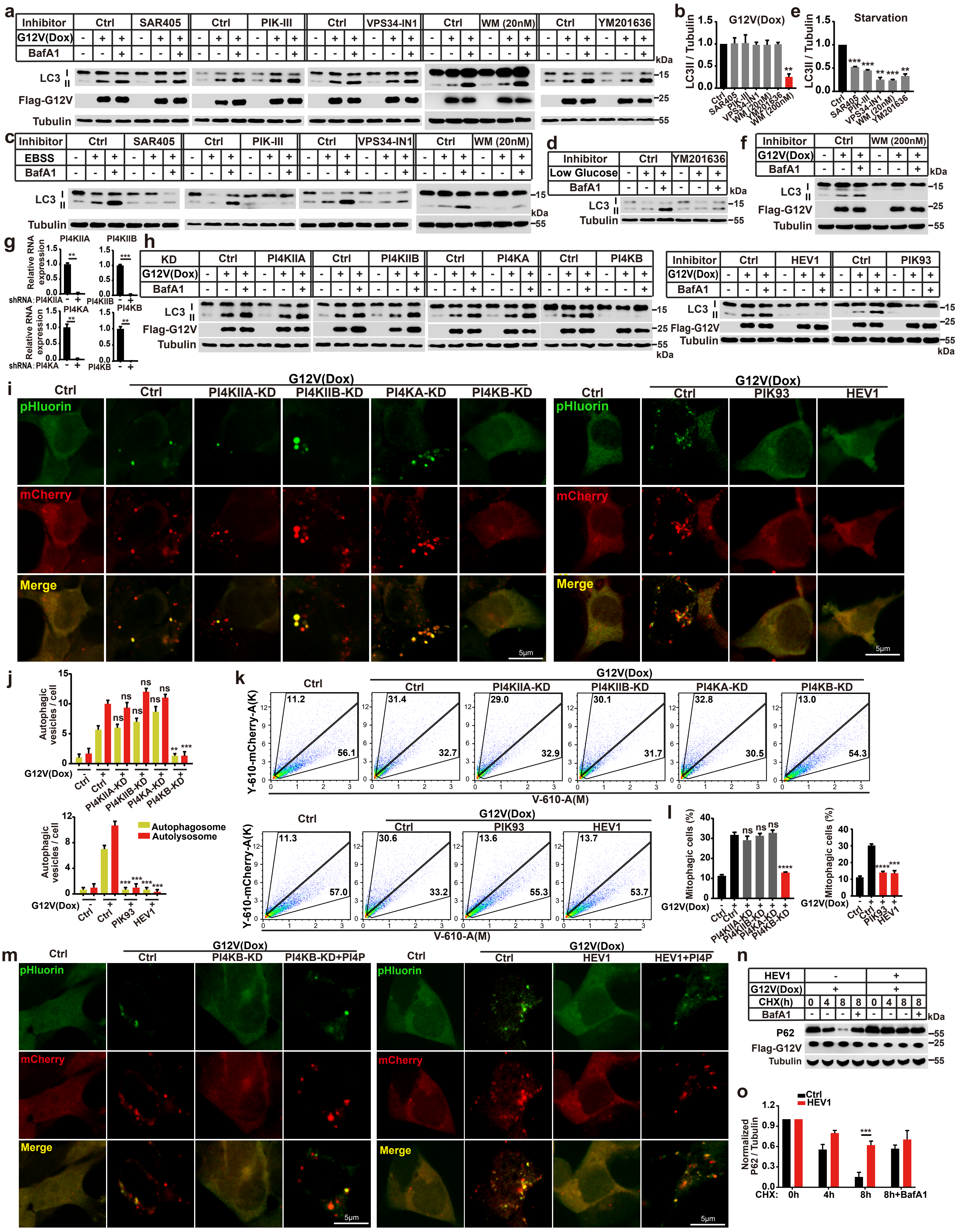

a
Inhibitor
Ctrl
SAR405
PIK-III
VPS34-IN1
WM (20nM)
YM201636
G12V(Dox)
BafA1
kDa
LC3 I II
Flag-G12V
Tubulin
-15
-25
-55
b
G12V(Dox)
LC3II / Tubulin
WM (200nM)
e
Starvation
c
EBSS
d
Low Glucose
f
WM (200nM)
g
Relative RNA expression
shRNA:PI4KIIA
shRNA:PI4KA
PI4KIIA
PI4KIIB
PI4KA
PI4KB
h
KD
HEV1
PIK93
i
pHluorin
mCherry
Merge
PI4KIIA-KD
PI4KIIB-KD
PI4KA-KD
PI4KB-KD
5μm
j
Autophagic vesicles / cell
Autophagosome
Autolysosome
ns
k
Y-610-mCherry-A(K)
V-610-A(M)
11.2
56.1
31.4
32.7
29.0
32.9
30.1
31.7
32.8
30.5
13.0
54.3
11.3
57.0
30.6
33.2
13.6
55.3
13.7
53.7
l
Mitophagic cells (%)
m
PI4KB-KD+PI4P
HEV1+PI4P
n
CHX(h)
P62
o
Normalized P62 / Tubulin
CHX:
0h
4h
8h
8h+BafA1

Figure. S3 The PI3K and PI5K complexes do not regulate RINCAA, but regulate the starvation-induced autophagy

a. Immunoblot analysis of LC3 lipidation in KRAS(G12V) and control cells treated with SAR405 (40 nM), PIK-III (40 nM), VPS34-IN1 (40 nM), Wortmannin (20 nM), or YM201636 (100 nM) in the absence or presence of 500 nM Bafilomycin A1 for 1.5 h.

b. Quantification of results shown in **a** (mean ± SEM). Three independent experiments were performed for the statistical analysis (two-tailed t-test). **, $P < 0.01$.

c. Immunoblot analysis of LC3 lipidation of cells treated with SAR405 (40 nM), PIK-III (40 nM), VPS34-IN1 (40 nM), or Wortmannin (20 nM) in starvation-induced autophagy by EBSS treatment in the absence or presence of 500 nM Bafilomycin A1 for 1.5 h.

d. Immunoblot analysis of LC3 lipidation of cells treated with YM201636 (100 nM) in low glucose-induced autophagy in the absence or presence of 500 nM Bafilomycin A1 for 1.5 h.

e. Quantification of results shown in **c, d** (mean ± SEM). Three independent experiments were performed for the statistical analysis (two-tailed t-test). **, $P < 0.01$; ***, $P < 0.001$.

f. Immunoblot analysis of LC3 lipidation in KRAS(G12V) and control cells treated with Wortmannin (200 nM) in the absence or presence of 500 nM Bafilomycin A1 for 1.5h.

g. Relative RNA expression of cells transfected with control or shRNAs against PI4KIIA, PI4KIIB, PI4KA or PI4KB (mean ± SEM). Three independent experiments were performed for the statistical analysis (two-tailed t-test). **, $P < 0.01$; ***, $P < 0.001$.

h. Immunoblot analysis of LC3 lipidation in KRAS(G12V) and control cells with Knockdown of PI4Ks or treatment of PI4KB inhibitors in the absence or presence of 500 nM Bafilomycin A1 for 1.5 h.

i. Immunofluorescence of KRAS(G12V) and control cells expressing mCherry-pHluorin-LC3B with Knockdown of PI4Ks or treatment of PI4KB inhibitors.

j. Quantification of the results in **i** (mean ± SEM). Three independent experiments (50 cells for each group/experiment) were performed for the statistical analysis (two-tailed t-test). **, $P < 0.01$; ***, $P < 0.001$.

k. FACS analysis of control and KRAS(G12V) cells co-expressing mt-Keima and Parkin with control or knockdown of PI4Ks using V610 and Y610-mCherry detectors (Beckman CytoFLEX LX). The FACS results are representative of at least Three independent independent experiments.

l. Quantification of results shown in **k**. The percentage of cells with mitophagy based on Y610-mCherry/V610. Data are represented as mean ± SEM. Three independent experiments were performed for the statistical analysis (two-tailed t-test). ***, $P < 0.001$; ****, $P < 0.0001$.

m. Immunofluorescence of HEK293T cells expressing mCherry-pHluorin-LC3B were rescued by PI4P with suppressed PI4KB by knockdown or treatment of the inhibitor. Representative cell images are shown. Scale bar sizes are indicated in the image.

n. Immunoblot analysis of turnover of P62 in CHX chase assay from KRAS(G12V) HEK293T cells treated with or without HEV1(10μM).

o. Quantification of the ratio of normalized P62 to tubulin with the 0h point set as 1.00 analyzed in **n** (mean ± SEM). Three independent experiments were performed for the statistical analysis (two-tailed t-test). ***, $P < 0.001$.
