# Supplementary material for: Oncogenic RAS induces a distinctive form of non-canonical autophagy mediated by the P38-ULK1-PI4KB axis
Source: Cell Res. 2025 Mar 7;35(6):399–422. doi: 10.1038/s41422-025-01085-9 (PMC12134136; doi:10.1038/s41422-025-01085-9)
Supplement: Supplementary file 4 — Fig. S4 [file 41422_2025_1085_MOESM4_ESM.pdf]

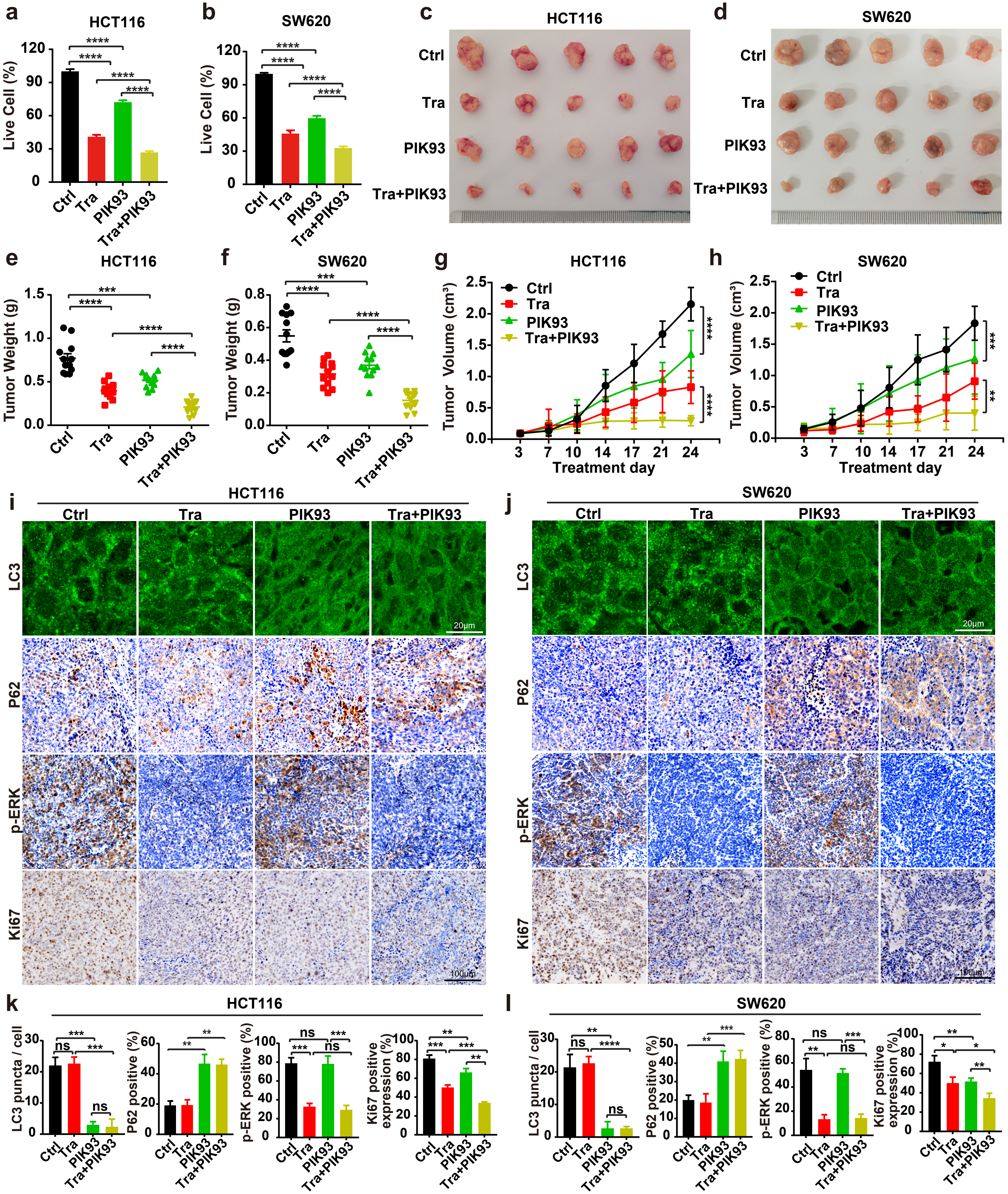

a
HCT116
Live Cell (%)
Ctrl
Tra
PIK93
Tra+PIK93
b
SW620
c
HCT116
d
SW620
e
HCT116
Tumor Weight (g)
f
SW620
g
HCT116
Tumor Volume (cm³)
Treatment day
h
SW620
i
HCT116
LC3
P62
p-ERK
Ki67
20μm
100μm
j
SW620
k
HCT116
LC3 puncta / cell
P62 positive (%)
p-ERK positive (%)
Ki67 positive expression (%)
l
SW620

Figure. S4 PI4KB inhibition slows xenograft tumors with RAS mutation

(a, b) HCT116 and SW620 cells were treated with trametinib (100 nM) and PIK93 (1 μM) for 96 h, and the cell viability was analyzed (mean ± SEM). Three independent experiments were performed for the statistical analysis (two-tailed t-test). ****, $P < 0.0001$.

(c, d) Images of xenograft tumors of HCT116 and SW620 cells from mice treated with: (1) vehicle (Ctrl); (2) Trametinib (Tra); (3) PIK93; or (4) the combination of both (Tra+PIK93) (n = 12 mice (one tumor/mice) in each group). The tumors were removed and photographed after 24-day treatment.

(e, f) Weights of the xenograft tumors in (**c, d**) (means ± SD). The statistical analysis was performed by two-tailed t-test. *, $P < 0.05$; **, $P < 0.01$; ****, $P < 0.0001$.

(g, h) The growth curve of the xenograft tumors in (**c, d**) (means ± SD). Statistical analysis was performed by two-way-ANOVA; ****, $P < 0.0001$.

(i, j) Immunofluorescence and immunohistochemical analysis of the sections of xenograft tumors in (**c, d**). Sections were stained with antibody against LC3, p-ERK1/2 or Ki67, as indicated. Scale bar sizes are indicated in the image.

(k, l) Statistical analysis of the numbers of LC3 puncta, p-ERK positive rates, and levels of Ki67 expression in (**I, J**) (means ± SD). Statistical analysis was performed by two-tailed t-test; *, $P < 0.05$; **, $P < 0.01$; ***, $P < 0.001$; ****, $P < 0.0001$.
