# Supplementary material for: Oncogenic RAS induces a distinctive form of non-canonical autophagy mediated by the P38-ULK1-PI4KB axis
Source: Cell Res. 2025 Mar 7;35(6):399–422. doi: 10.1038/s41422-025-01085-9 (PMC12134136; doi:10.1038/s41422-025-01085-9)
Supplement: Supplementary file 5 — Fig. S5 [file 41422_2025_1085_MOESM5_ESM.pdf]

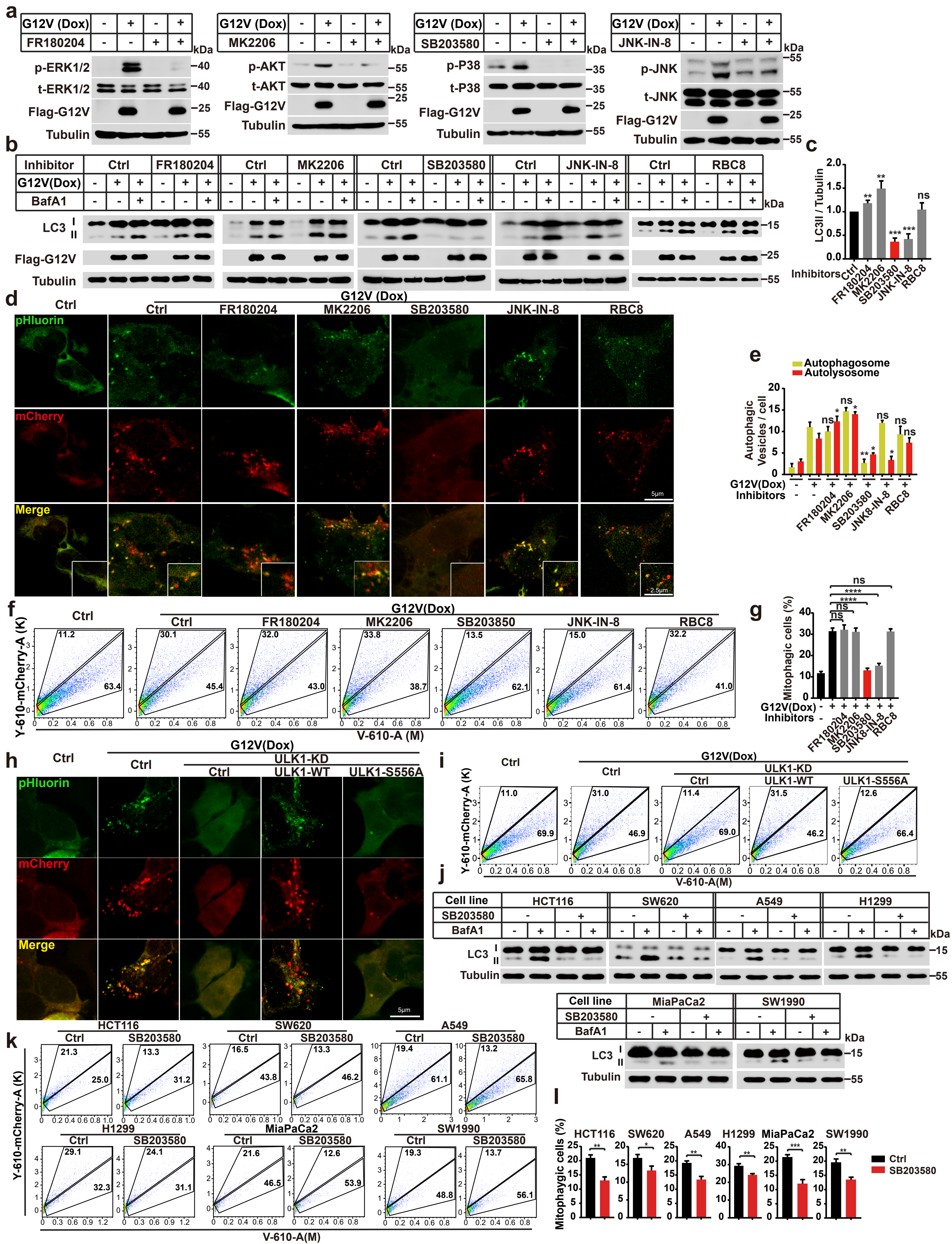

a
G12V (Dox)
FR180204
MK2206
SB203580
JNK-IN-8
kDa
p-ERK1/2
t-ERK1/2
Flag-G12V
Tubulin
p-AKT
t-AKT
p-P38
t-P38
p-JNK
t-JNK
b
Inhibitor
Ctrl
RBC8
G12V(Dox)
BafA1
LC3 I II
c
LC3II / Tubulin
Inhibitors
d
G12V (Dox)
pHluorin
mCherry
Merge
5μm
2.5μm
e
Autophagosome
Autolysosome
Autophagic Vesicles / cell
JNK8-IN-8
f
G12V(Dox)
SB203850
Y-610-mCherry-A (K)
V-610-A (M)
g
Mitophagic cells (%)
h
ULK1-KD
ULK1-WT
ULK1-S556A
i
j
Cell line
HCT116
SW620
A549
H1299
MiaPaCa2
SW1990
k
l
Mitophagic cells (%)
Ctrl
SB203580

Figure. S5 The P38-pathway is required for RINCAA

a. Immunoblot analysis of levels of p-ERK, p-AKT, p-P38 or p-JNK in control and KRAS(G12V)-cells treated with FR180204 (10 μM), MK2206 (10 μM), SB203580 (10 μM) or JNK-IN-8 (10 μM).

b. Immunoblot analysis of LC3 lipidation of the KRAS(G12V)-cells treated with FR180204 (10 μM), MK2206 (10 μM), SB203580 (10 μM), or RBC8 (10 μM) in the absence or presence of 500 nM Bafilomycin A1 for 1.5 h.

c. Quantification of the results in **b** (mean ± SEM). Three independent experiments were performed for the statistical analysis (two-tailed t-test). **, $P < 0.01$; ***, $P < 0.001$.

d. Immunofluorescence of HEK293T cells expressing mCherry-pHluorin-LC3B treated with FR180204 (10 μM), MK2206 (10 μM), SB203580 (10 μM), or RBC8 (10 μM). Representative cell images are shown. Scale bar sizes are indicated in the image.

e. Quantification of the yellow (RFP^+GFP^+) and Red (RFP^+GFP^-) LC3 puncta in **d**. Data are represented as mean ± SEM. Three independent experiments (50 cells for each group/experiment) were performed for the statistical analysis (two-tailed t-test). *, $P < 0.05$; **, $P < 0.01$.

f. FACS analysis of control and KRAS(G12V) cells co-expressing mt-Keima and Parkin treated with FR180204 (10 μM), MK2206 (10 μM), SB203580 (10 μM), or RBC8 (10 μM) using V610 and Y610-mCherry detectors (Beckman CytoFLEX LX). The FACS results are representative of at least Three independent independent experiments.

g. Quantification of results shown in **f**. The percentage of cells with mitophagy based on Y610-mCherry/V610. Data are represented as mean ± SEM. Three independent experiments were performed for the statistical analysis (two-tailed t-test). ****, $P < 0.0001$.

h. Immunofluorescence of HEK293T cells expressing mCherry-pHluorin-LC3B treated with FR180204 (10 μM), MK2206 (10 μM), SB203580 (10 μM), or RBC8 (10 μM). Representative cell images are shown. Scale bar sizes are indicated in the image.

i. FACS analysis of control and KRAS(G12V) cells co-expressing mt-Keima and Parkin. To analyze the rescue effects of WT-ULK1 and ULK1-S556A on the KRAS(G12V)-induced mitophagy. The percentage of cells with mitophagy based on Y610-mCherry/V610. The FACS results are representative of at least Three independent

independent experiments.

j. Immunoblot analysis of LC3 lipidation of the different cancer cell lines treated with or without SB203580 (10 μM) in the absence or presence of 500 nM Bafilomycin A1 for 1.5 h.

k. FACS analysis of the different cancer cell lines co-expressing mt-Keima and Parkin treated with or without SB203580 (10 μM). The percentage of cells with mitophagy was calculated based on Y610-mCherry/V610. The FACS results are representative of at least Three independent independent experiments.

l. Quantification of results shown in **k**. The percentage of cells with mitophagy based on Y610-mCherry/V610. Data are represented as mean ± SEM. Three independent experiments were performed for the statistical analysis (two-tailed t-test). ****, $P < 0.0001$.
