# Supplementary material for: Oncogenic RAS induces a distinctive form of non-canonical autophagy mediated by the P38-ULK1-PI4KB axis
Source: Cell Res. 2025 Mar 7;35(6):399–422. doi: 10.1038/s41422-025-01085-9 (PMC12134136; doi:10.1038/s41422-025-01085-9)
Supplement: Supplementary file 6 — Fig. S6 [file 41422_2025_1085_MOESM6_ESM.pdf]

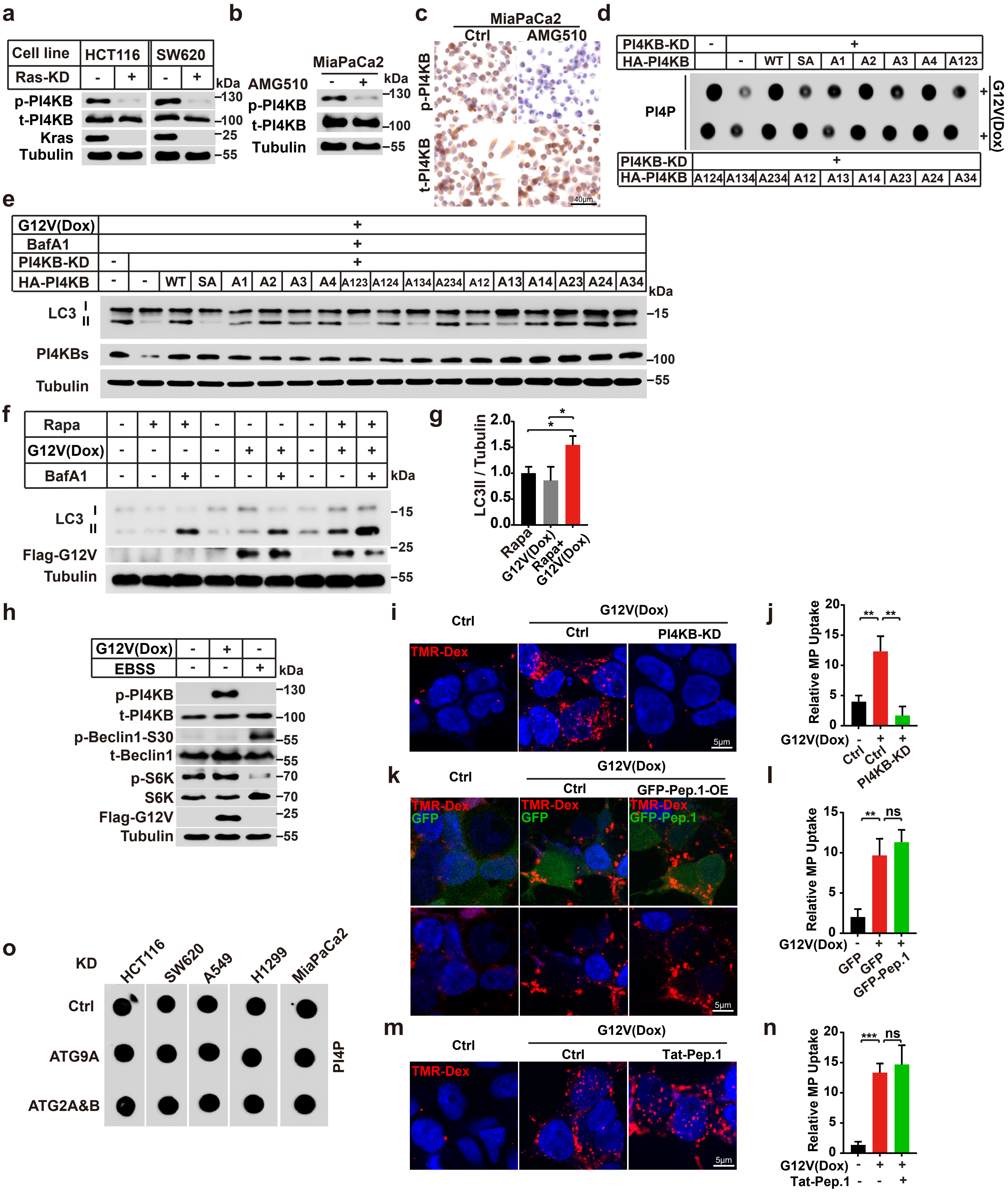

a
Cell line
HCT116
SW620
Ras-KD
p-PI4KB
t-PI4KB
Kras
Tubulin
kDa
b
MiaPaCa2
AMG510
p-PI4KB
t-PI4KB
Tubulin
c
MiaPaCa2
Ctrl
AMG510
p-PI4KB
t-PI4KB
40μm
d
PI4KB-KD
HA-PI4KB
WT
SA
A1
A2
A3
A4
A123
PI4P
G12V(Dox)
A124
A134
A234
A12
A13
A14
A23
A24
A34
e
G12V(Dox)
BafA1
PI4KB-KD
HA-PI4KB
LC3
PI4KBs
Tubulin
f
Rapa
G12V(Dox)
BafA1
LC3
Flag-G12V
Tubulin
g
LC3II / Tubulin
Rapa
G12V(Dox)
Rapa+ G12V(Dox)
h
G12V(Dox)
EBSS
p-PI4KB
t-PI4KB
p-Beclin1-S30
t-Beclin1
p-S6K
S6K
Flag-G12V
Tubulin
i
Ctrl
G12V(Dox)
Ctrl
PI4KB-KD
TMR-Dex
5μm
j
Relative MP Uptake
G12V(Dox)
Ctrl
Ctrl
PI4KB-KD
k
Ctrl
G12V(Dox)
Ctrl
GFP-Pep.1-OE
TMR-Dex
GFP
GFP-Pep.1
5μm
l
Relative MP Uptake
G12V(Dox)
GFP
GFP
GFP-Pep.1
ns
m
Ctrl
G12V(Dox)
Ctrl
Tat-Pep.1
TMR-Dex
5μm
n
Relative MP Uptake
G12V(Dox)
Tat-Pep.1
ns
o
KD
HCT116
SW620
A549
H1299
MiaPaCa2
Ctrl
ATG9A
ATG2A&B
PI4P

Figure. S6 Validation of the PI4KB-peptide 1 phosphorylation antibody, and pinpointing the phosphorylation of PI4KB

a. Immunoblot analysis of the PI4KB phosphorylation by the antibody against p-PI4KB in HCT116 and SW620 cells with or without RAS knockdown.

b. Immunoblot analysis of the PI4KB phosphorylation by the antibody against p-PI4KB in MiaPaCa-2 cells treated with or without with 10 μM AMG510 for 1.5 h.

c. Immunohistochemical analysis of the sections of MiaPaCa-2 cells treated with or without with 10 μM AMG510 for 1.5 h. Sections were stained with antibody against PI4KB and p-PI4KB as indicated. Scale bar sizes are indicated in the image.

d. Dot blot analysis of the effect of PI4KB mutants on the KRAS(G12V)-induced PI4P generation in HEK293T cells.

e. Immunoblot analysis of the rescue effect of PI4KB-WT, PI4KB-SA and PI4KB mutants on KRAS(G12V)-induced LC3 lipidation after knockdown PI4KB in HEK293T cells in the absence or presence of 500 nM Bafilomycin A1 for 1.5 h.

f. Immunoblot analysis of the LC3 lipidation of the cell lysates from the control or KRAS(G12V) cells treated with 2 μM Rapamycin for 1.5 h in the absence or presence of 500 nM Bafilomycin A1 for 1.5 h.

g. Quantification of the results in **f** (mean ± SEM). Three independent experiments were performed for the statistical analysis (two-tailed t-test). *, $P < 0.05$.

h. Immunoblot analysis of the changes of the level of p-PI4KB, p-Beclin1 and p-S6K in the KRAS(G12V) and starvation HEK293T cells.

i. Macropinocytosis visualization using TMR-Dex in control and KRAS(G12V) HEK293T cells with or without PI4KB knockdown. Representative cell images are shown. Scale bar sizes are indicated in the image.

j. Quantification of macropinocytosis in **i**. Data are represented as mean ± SEM. Three independent experiments (50 cells for each group/experiment) were performed for the statistical analysis (two-tailed t-test). **, $P < 0.01$.

k. Macropinocytosis visualization using TMR-Dex in control and KRAS(G12V) HEK293T cells with or without GFP-Peptide1 (GFP-Pep.1) overexpression. Representative cell images are shown. Scale bar sizes are indicated in the image.

l. Quantification of macropinocytosis in **k**. Data are represented as mean ± SEM. Three

independent experiments (50 cells for each group/experiment) were performed for the statistical analysis (two-tailed t-test). **, $P < 0.01$.

m. Macropinocytosis visualization using TMR-Dex (1 mg/mL) in control and KRAS(G12V) HEK293T cells with or without Tat-Peptide1 (Tat-Pep.1, 25 μM) treatment for 2 h. Representative cell images are shown. Scale bar sizes are indicated in the image.

n. Quantification of macropinocytosis in **m**. Data are represented as mean ± SEM. Three independent experiments (50 cells for each group/experiment) were performed for the statistical analysis (two-tailed t-test). ***, $P < 0.001$.
