# Supplementary material for: Oncogenic RAS induces a distinctive form of non-canonical autophagy mediated by the P38-ULK1-PI4KB axis
Source: Cell Res. 2025 Mar 7;35(6):399–422. doi: 10.1038/s41422-025-01085-9 (PMC12134136; doi:10.1038/s41422-025-01085-9)
Supplement: Supplementary file 8 — Table. S1 [file 41422_2025_1085_MOESM8_ESM.docx]

| **Supplementary information, Table S1 MS of p-PI4KB.** The peptides identified were listed in the table including name, sequence, modifications and percent of phosphorylated peptides. |
| --- |

| Name | Sequence | Modifications | % of phosphorylated peptides | | |
| --- | --- | --- | --- | --- | --- |
|  |  |  | Con | ULK | ULK+IN |
| Peptide 1 | KRELPSLSPAPDTGLSPSK | S6(Phospho); S14(Phospho) | 0.35 | 0.61 | 0.14 |
| Peptide 2 | SKSDATASISLSSNLKR | S3(Phospho) | 0.55 | 0.45 | 0.46 |
| Peptide 3 | RTAsNPKVENEDEPVR | T2(Phospho); S4(Phospho) | 1.00 | 1.00 | 1.00 |
| Peptide 4 | STRsVENLPEcGITHEQR | S1(Phospho); S4(Phospho) | 1.00 | 1.00 | 1.00 |
| Peptide 5 | RLSEQLAHTPTAFKR | S2(Phospho); S3(Phospho); S4(Phospho) | 0.48 | 0.61 | 0.77 |
