# Supplementary material for: Oncogenic RAS induces a distinctive form of non-canonical autophagy mediated by the P38-ULK1-PI4KB axis
Source: Cell Res. 2025 Mar 7;35(6):399–422. doi: 10.1038/s41422-025-01085-9 (PMC12134136; doi:10.1038/s41422-025-01085-9)
Supplement: Supplementary file 9 — Table. S2 [file 41422_2025_1085_MOESM9_ESM.docx]

**Supplementary information, Table S2 Antibodies used in this study.** Antibodies used in this study were listed in the table including name, source and identifier.

| **Antibodies Source Identifier** | | | |
| --- | --- | --- | --- |
| Mouse monoclonal anti-FLAG | Sigma | | Cat# F3165;  RRID: AB_259529 |
| Rabbit monoclonal anti-FLAG | | CST | Cat# 14793;  RRID: AB_2572291 |
| Rat monoclonal anti-FLAG | | Novus | Cat# NBP1-06712SS;  RRID: AB_1625982 |
| Rabbit monoclonal anti-HA | | CST | Cat# 3724;  RRID: AB_1549585 |
| Mouse monoclonal anti-Myc | | CST | Cat# 2276;  RRID: AB_331783 |
| Rabbit polyclonal anti-LC3 (WB) | | ABclonal | Cat# A19665;  RRID: AB_2862723 |
| Rabbit polyclonal anti-LC3 (IF) | | MBL | Cat# PM036;  RRID: AB_2274121 |
| Mouse monoclonal anti-LC3 (IF) | | MBL | Cat# M152-3;  RRID: AB_1279144 |
| Rabbit polyclonal anti-LC3 (IHC) | | ABcepta | Cat# AP1802a,  RRID: AB_2137695 |
| Mouse monoclonal anti-β-Tubulin | | Zen Bioscience | Cat# 200608;  RRID: AB_2722706 |
| Rabbit monoclonal anti-ULK1 | | CST | Cat# 8054;  RRID: AB_11178668 |
| Rabbit monoclonal anti-Phospho-ULK1 (Ser555) | | CST | Cat#5869;  RRID: AB_10707365 |
| Rabbit polyclonal anti-ULK1 (Ser556) conjugated to Biotin  Rabbit monoclonal anti-Phospho-ULK1 (Ser317)  Rabbit monoclonal anti-Phospho-ULK1 (Ser757)  Rabbit monoclonal anti-Phospho-Becline-1(Ser30) | | Biorbyt  CST  CST  CST | Cat# orb502261  Cat#6887S;  RRID: AB_10831845  Cat#14202S;  RRID: AB_2665508  Cat#35955S; |
| Rabbit polyclonal anti-RB1CC1(FIP200) | | Proteintech | Cat# 17250-1-AP;  RRID: AB_10666428 |
| Mouse monoclonal anti-ATG5 | | MBL | Cat# M153-3;  RRID: AB_1278760 |
| Mouse monoclonal anti-ATG16L1 | | MBL | Cat# M150-3;  RRID: AB_1278758 |
| Mouse monoclonal anti-WIPI2 | | Bio-Rad | Cat# MCA5780GA;  RRID: AB_10845951 |
| Rabbit polyclonal anti-mCherry | | This paper | N/A |
| Rabbit polyclonal anti-Beclin1 | | Sigma | Cat# PRS3613;  RRID: AB_1845329 |
| Rabbit polyclonal anti-ATG14 | | MBL | Cat# PD026;  RRID: AB_1953054 |
| Rabbit polyclonal anti-ATG9A | | MBL | Cat# PD042;  RRID: AB_2714019 |
| Rabbit monoclonal anti-ATG9A | | CST | Cat# 13509;  RRID: AB_2798241 |
| Rabbit polyclonal anti-ATG2A | | MBL | Cat# PD041;  RRID: AB_2810871 |
| Rabbit polyclonal anti-ATG2B | | Sigma | Cat# HPA019665;  RRID: AB_2274278 |
| Mouse anti-PI4P | | Echelon | Cat# Z-P004;  RRID: AB_11127796 |
| Rabbit polyclonal anti-PI4KB | | Abcam | Cat# Ab134756 |
| Rabbit monoclonal anti-GFP (WB) | | CST | Cat# 2956;  RRID: AB_1196615 |
| Rabbit monoclonal anti-ERK | | CST | Cat# 4695;  RRID: AB_390779 |
| Rabbit monoclonal anti-Phospho-ERK | | CST | Cat# 4370;  RRID: AB_2315112 |
| Rabbit monoclonal anti-AKT | | CST | Cat# 4691;  RRID: AB_915783 |
| Rabbit monoclonal anti-Phospho-AKT | | CST | Cat# 4060;  RRID: AB_2315049 |
| Rabbit monoclonal anti-P38 | | CST | Cat# 8690;  RRID: AB_10999090 |
| Rabbit monoclonal anti-Phospho-P38 | | CST | Cat# 4511;  RRID: AB_213968 |
| Rabbit polyclonal anti-JNK | | CST | Cat# 9252;  RRID: AB_2250373 |
| Rabbit polyclonal anti-Phospho-JNK | | CST | Cat# 4668;  RRID: AB_823588 |
| Rabbit polyclonal anti-Ki67 | | Abcam | Cat# ab15580,  RRID: AB_443209 |
| Rabbit polyclonal anti-Ribophorin1(RPN1) | | Dr. Randy Schekman | N/A |
| Rabbit polyclonal anti-Phospho-PI4KB | | N/A | N/A |
| Rabbit polyclonal anti-STX17 | | Proteintech | Cat# 17815-1-AP,  RRID: AB_2255542 |
| Rabbit polyclonal anti-SNAP29 | | Proteintech | Cat# 12704-1-AP,  RRID: AB_2192340 |
| Rabbit polyclonal anti-VAMP8 | | Proteintech | Cat# 15546-1-AP,  RRID: AB_2878150 |
| Rabbit polyclonal anti-KRAS | | ABclonal | Cat# A1190,  RRID: AB_2758846 |
| Rabbit polyclonal anti-NRAS | | ABclonal | Cat# A7566  RRID: AB_2770660 |
| Rabbit polyclonal anti-p70 S6K | | CST | Cat# 2708S  RRID: AB_390722 |
| Rabbit polyclonal anti-p-p70 S6K | | CST | Cat# 9025S  RRID: AB_2734746 |
