# Supplementary material for: Oncogenic RAS induces a distinctive form of non-canonical autophagy mediated by the P38-ULK1-PI4KB axis
Source: Cell Res. 2025 Mar 7;35(6):399–422. doi: 10.1038/s41422-025-01085-9 (PMC12134136; doi:10.1038/s41422-025-01085-9)
Supplement: Supplementary file 10 — Table. S3 [file 41422_2025_1085_MOESM10_ESM.docx]

| **Supplementary information, Table S3 Oligonucleotides used in this study.** Oligonucleotides for siRNAs, shRNAs and primers used in this study were listed in the table including name and sequence. | |
| --- | --- |
| **Oligos for siRNAs** | |
| ULK1 siRNA target sequence-1: | CGCGCGGTACCTCCAGAGCAA |
| ULK1 siRNA target sequence-2: | TGCCCTTTGCGTTATATTGTA |
| FIP200 siRNA target sequence-1: | CTGGGACGGATACAAATCCAA |
| FIP200 siRNA target sequence-2: | ACGCAAATCAGTTGATGATTA |
| ATG5 siRNA target sequence-1: | AACCTTTGGCCTAAGAAGAAA |
| ATG5 siRNA target sequence-2: | CTAGGAGATCTCCTCAAAGAA |
| ATG5 siRNA target sequence-3: | AAGACTTACCGGACCACTGAA |
| ATG5 siRNA target sequence-4: | CATCATAGCTTTATTACTCTA |
| ATG16L1 siRNA target sequence-1: | GGAGATCATCCTGCAGTATAA |
| ATG16L1 siRNA target sequence-2: | CACGAGATAAGTCCCGGACAT |
| ATG16L1 siRNA target sequence-3: | CTCCCGTGATGACTTGCTAAA |
| ATG16L1 siRNA target sequence-4: | CAGGATCCAGTTGCAATGATA |
| WIPI2 siRNA target sequence-1: | ACACGATTCTGGCTGTGAA |
| WIPI2 siRNA target sequence-2: | TGACGCAAGTGGAACTAAA |
| WIPI2 siRNA target sequence-3: | CGCTGTCAATCAACAACGA |
| Beclin1 siRNA target sequence-1: | GAGGATGACAGTGAACAGTTA |
| Beclin1 siRNA target sequence-2: | TGGACAGTTTGGCACAATCAA |
| Beclin1 siRNA target sequence-3: | AGGGTCTAAGACGTCCAACAA |
| Beclin1 siRNA target sequence-4: | ACCGACTTGTTCCTTACGGAA |
| ATG14 siRNA target sequence-1: | CTCGGTGACCTCCTGGTTTAA |
| ATG14 siRNA target sequence-2: | TTGGATTAGCCTCCCTAACAA |
| ATG14 siRNA target sequence-3: | CTGCATACCCTCAGGAATCTA |
| ATG14 siRNA target sequence-4: | CCGGGAGAGGTTTATCGACAA |
| ATG2A siRNA target sequence-1: | Dr, M. Zhang |
| ATG2B siRNA target sequence-2: | Dr, M. Zhang |
| PI4KB siRNA target sequence-1: | GCGACATGTTCAACTACTA |
| PI4KB siRNA target sequence-2: | GCACCATTCGAAACCTCAA |
| PI4KB siRNA target sequence-3: | CTTGCTCGATTACTTCCTA |
| **Oligos for shRNAs** | |
| Ctrl shRNA target sequence: | CAACAAGATGAAGAGCACCAA |
| ULK1shRNA target sequence-1: | GCCCTGGATACGTCTTGTAAT |
| ULK1shRNA target sequence-2: | GCCCTTTGCGTTATATTGTAT |
| PI4KIIA shRNA target sequence-1: | CCCTAACTTCGTCAAGGACTT |
| PI4KIIA shRNA target sequence-2: | CCTCTTCCTGAGAACACTAAC |
| PI4KIIB shRNA target sequence-1: | GACATGAACTTTGTGCAAGAT |
| PI4KIIB shRNA target sequence-2: | CCTGATGAATGGAGAGCATAT |
| PI4KA shRNA target sequence-1: | ACGACATGATCCAGTACTATC |
| PI4KA shRNA target sequence-2: | CAAGGCTGGATCAACACATAC |
| PI4KB shRNA target sequence-1: | GCAAGAAACACGAAGGATCAT |
| PI4KB shRNA target sequence-2: | CCACAGGCCATCCTCTTATT |
| VPS13B shRNA target sequence-1: | GCCTTACTCAACCTTCTGATA |
| VPS13B shRNA target sequence-2: | ATGGTACTCGCGCAGAATTTA |
| VPS13C shRNA target sequence-1: | CGGACAAAGTTAATCCAACAA |
| VPS13C shRNA target sequence-2: | GCAGTAACTAATGCCCTAAAT |
| VPS13D shRNA target sequence-1: | CCCAGAAAGTTCCAGATCAAA |
| VPS13D shRNA target sequence-2: | GCCAGATTTGACTTCAAGAAA |
| STX17 shRNA target sequence-1: | CGATCCAATATCCGAGAAATT |
| STX17 shRNA target sequence-2: | TTGTTATCAGATAGCGAAATC |
| SNAP29 shRNA target sequence-1: | CCAGAAACACATCAATAGCAT |
| SNAP29 shRNA target sequence-2: | ACAACCAAAGTGGACAAGTTA |
| VAMP8 shRNA target sequence-1: | CTTGGAACATCTCCGCAACAA |
| VAMP8 shRNA target sequence-2: | GATTGTCCTTATCTGCGTGAT |
| TMEM41B shRNA target sequence-1: | GTTGAACGTCATAGAGAACAT |
| TMEM41B shRNA target sequence-2: | GTTTCCTGGAACTCAATATTT |
| VMP1 shRNA target sequence-1: | GCAATGAACAAGGAACATCAT |
| VMP1 shRNA target sequence-2: | CGATGCAATCCACCTTGTGTT |
| **Oligos for Primers** | |
| PI4KIIA forward primer: | TTGTCCTTAACCAGGGCTATCT |
| PI4KIIA reverse primer: | GTACGGGGAACAATGTTGAGT |
| PI4KIIB forward primer: | ACCCAAATCAGAAGAGCCTTATG |
| PI4KIIB reverse primer: | CAAGGGCAGCAGACCTTATG |
| PI4KA forward primer: | AACCTGGACATAACTGTCGGC |
| PI4KA reverse primer: | GAGCCCCGGTTGGTTTTCTT |
| PI4KB forward primer: | CCTGCTCAACCATAAGCTCCC |
| PI4KB reverse primer: | AGTTTTCTACGGACCTCGTACT |
| ACTIN forward primer: | CATGTACGTTGCTATCCAGGC |
| ACTIN reverse primer: | CTCCTTAATGTCACGCACGAT |
| VPS13B forward primer: | ATCGTCCTTTCCGTCAATATCAC |
| VPS13B reverse primer: | CAGCACCAAATCAGTTGCAGA |
| VPS13C forward primer: | ACTTGATAGTCTTAGCGCCTACT |
| VPS13C reverse primer: | ATCCAGTTTGGGCGTTTTGAG |
| VPS13D forward primer: | TACCGCCTCCGTAGTTACAAG |
| VPS13D reverse primer: | GTAAAGTGCAATCGACATCCCA |
| TMEM41B forward primer: | CAGAGTCGCCGAACGATCG |
| TMEM41B reverse primer: | CCTAGAGCCTTGGCATCATCCA |
| VMP1 forward primer: | GACCAGAGACGTGTAGCAATG |
| VMP1 reverse primer: | ACAATGCTTTGACGATGCCATAA |
